# Quantifying the Metabolic Signature of Multiple Sclerosis by *in vivo* Proton Magnetic Resonance Spectroscopy: Current Challenges and Future Outlook in the Translation From Proton Signal to Diagnostic Biomarker

**DOI:** 10.3389/fneur.2019.01173

**Published:** 2019-11-15

**Authors:** Kelley M. Swanberg, Karl Landheer, David Pitt, Christoph Juchem

**Affiliations:** ^1^Department of Biomedical Engineering, Columbia University Fu Foundation School of Engineering and Applied Science, New York, NY, United States; ^2^Department of Neurology, Yale University School of Medicine, New Haven, CT, United States; ^3^Department of Radiology, Columbia University College of Physicians and Surgeons, New York, NY, United States

**Keywords:** multiple sclerosis, *in vivo* proton magnetic resonance spectroscopy 1H-MRS, biomarker, longitudinal relaxation T1, transverse relaxation T2, spectroscopic baseline, absolute quantification, macromolecules

## Abstract

Proton magnetic resonance spectroscopy (^1^H-MRS) offers a growing variety of methods for querying potential diagnostic biomarkers of multiple sclerosis in living central nervous system tissue. For the past three decades, ^1^H-MRS has enabled the acquisition of a rich dataset suggestive of numerous metabolic alterations in lesions, normal-appearing white matter, gray matter, and spinal cord of individuals with multiple sclerosis, but this body of information is not free of seeming internal contradiction. The use of ^1^H-MRS signals as diagnostic biomarkers depends on reproducible and generalizable sensitivity and specificity to disease state that can be confounded by a multitude of influences, including experiment group classification and demographics; acquisition sequence; spectral quality and quantifiability; the contribution of macromolecules and lipids to the spectroscopic baseline; spectral quantification pipeline; voxel tissue and lesion composition; *T*_1_ and *T*_2_ relaxation; B_1_ field characteristics; and other features of study design, spectral acquisition and processing, and metabolite quantification about which the experimenter may possess imperfect or incomplete information. The direct comparison of ^1^H-MRS data from individuals with and without multiple sclerosis poses a special challenge in this regard, as several lines of evidence suggest that experimental cohorts may differ significantly in some of these parameters. We review the existing findings of *in vivo* ^1^H-MRS on central nervous system metabolic abnormalities in multiple sclerosis and its subtypes within the context of study design, spectral acquisition and processing, and metabolite quantification and offer an outlook on technical considerations, including the growing use of machine learning, by future investigations into diagnostic biomarkers of multiple sclerosis measurable by ^1^H-MRS.

## Introduction

*In vivo* proton magnetic resonance spectroscopy (^1^H-MRS) is a method that can estimate the concentrations of select small molecules in living tissue using electromagnetic waves to manipulate and monitor the behavior of hydrogen nuclear spins in a magnetic field. Since Felix Bloch of Stanford University performed in 1946 what is now considered to be the first *in vivo* MRS measurement, of his own finger (1), *in vivo* spectroscopy has developed considerably, now enabling the routine assessment of multiple small-molecule metabolites in tissues like the human skeletal muscle (2), liver (3), brain (4), heart (5), breast (6), bone (7), prostate (8), kidney (9), and, more recently, the spinal cord (10).

Despite the diagnostic potential suggested by the range of organs to which spectroscopy has been applied to safely and non-invasively investigate *in vivo* metabolism in the laboratory, its clinical utility remains limited. In the brain, ^1^H-MRS is currently used as an auxiliary to magnetic resonance imaging (MRI) for clinical decision-making in tumors, neonatal hypoxia and congenital metabolic disorders and leukoencephalopathies, pediatric traumatic brain injury, and infectious brain abscesses (11). Its application to other conditions is, however, minimal. This fact is reflected in reimbursement protocols published by the largest three health insurance providers in the United States, according to which ^1^H-MRS is “unproven and/or not medically necessary” (United Health Group) (12), “investigational and not medically necessary” for all non-oncological indications (Anthem) (13), and “experimental and investigational” for all but the characterization of brain tumors and other biopsy-eligible lesions (Aetna) (14). ^1^H-MRS thus currently represents a wealth of unpolished potential in the diagnostics of countless diseases currently evaluated by slower, more invasive, or otherwise riskier means.

Multiple sclerosis is one such disease. With more than 2.3 million patients worldwide and a prevalence that appears to be rising in the United States (15) and potentially among ethnic groups historically considered to be low-risk (16), multiple sclerosis is a chronic autoimmune condition that targets the central nervous system, rendering it one of the most common causes of neurological disability in young adults (17). Its diagnosis continues to depend on the subjective and uncertain (18) multifactorial synthesis of symptom self-report, neurological evaluation, and magnetic resonance imaging for central nervous system lesions, in addition to lumbar puncture for assay of oligoclonal bands in the cerebrospinal fluid (19). Diagnosis is further complicated by the presence of at least three disease phenotypes with distinct courses and expected response to commonly employed disease-modifying therapies: relapsing-remitting multiple sclerosis, secondary progressive multiple sclerosis, and primary progressive multiple sclerosis, differences among which will be detailed later.

Despite abundant evidence that multiple sclerosis affects numerous metabolites measurable by brain ^1^H-MRS, spectroscopy is not currently used as a first-line diagnostic tool for the disease. Translation from the metabolite signals acquired via ^1^H-MRS to clinically useable predictive biomarkers still awaits the development of techniques either to maximize the precision with which one can isolate one or two metabolic smoking guns, as in improved shimming or spectral editing, or to uncover a reproducible pattern of subtle disease effects on multiple metabolites, as in machine learning. Most likely, the successful development of multiple sclerosis diagnostic biomarkers from ^1^H-MRS data will represent a combination of the two efforts. Spectroscopic data inputs to disease classifiers may be normalized, dimensionally reduced, or otherwise transformed into uninterpretable arbitrary units and therefore need not represent physically descriptive measurements of absolute molarity to be clinically useful. Even metabolite signals in arbitrary units, however, must enable classification schemes that are both sensitive and reproducible. Increased within-group variance from the variable influence of extra-concentration factors like relaxation, for example, may reduce the sensitivity of a potential classifier. On the other hand, increased between-group variance from a confound that may not be generalizable to every sequence, such as diffusion weighting, can enable the development of a seemingly sensitive classifier in one specialized experiment—for instance, metabolite values referenced to differentially diffusion-weighted water signals—but obstruct the usefulness of its broad application to clinical decision-making unless the nature of this confound is precisely understood.

As will be discussed, spectroscopic quantification may encompass multiple sources of obfuscating within-group variance or misleading between-group variance. These can include unrepresentative or mismatched subject sampling (section Study Cohort Demographics), unconsidered effects of spectral acquisition and processing techniques (section Spectral Acquisition and Processing), and faulty evidentiary support for conversion factors used to translate between signal intensity and usable concentration values (section Metabolite Quantification). Some potential confounds to metabolite quantification by ^1^H-MRS, including atrophy index, voxel gray-white matter composition, diffusion weighting, metabolite *T*_1_ and *T*_2_ relaxation constants, and other MR-visible differences in tissue physiology may, when characterized and controlled, serve as useful diagnostic biomarkers in their own right. The present review focuses, however, on minimizing their influence on the primary utility of ^1^H-MRS as a measure of *in vivo* small molecule concentrations in the march toward a clinically useful diagnostic biomarker for multiple sclerosis.

## Potential Small-Molecule Diagnostic Biomarkers of Multiple Sclerosis Measurable by ^1^H-MRS

### Small Molecules Examined in ^1^H-MRS Investigations of Multiple Sclerosis

Proton spectroscopic analysis of multiple sclerosis has examined a variety of individual small molecules in living central nervous system tissue, including N-acetyl aspartate, creatine, choline, myoinositol, glutamate, glutamine, γ-aminobutyric acid (GABA), glutathione, and lactate. For reasons of difficult isolation from a single spectral dataset, some of these and other metabolites are often grouped under more general categories, like total creatine (creatine and phosphocreatine), total N-acetyl aspartate (N-acetyl aspartate or NAA plus N-acetyl aspartylglutamate or NAAG), total choline (choline, phosphocholine, and glycerophosphocholine), inositol (myoinositol and scylloinositol), or Glx (glutamate and glutamine). Since different authors exhibit varying precision in nomenclature, for simplicity in the present review the terms N-acetyl aspartate, creatine, and choline may refer to any subset of biomolecules listed above in the “total” definitions thereof. In addition to small-molecule metabolites comprising the principal peaks of a ^1^H-MRS spectrum, the less well-defined lipids and macromolecules constituting the broader background signatures of some sequences have also been examined for differences between individuals with and without multiple sclerosis.

By far the majority of ^1^H-MRS studies of multiple sclerosis examine one or all of the highest-amplitude signals on a standard ^1^H-MRS localizing sequence, from N-acetyl aspartate, creatine, and choline. A detailed treatment of the potential roles of each class of molecules in healthy and diseased brain is well beyond the scope of the present review. It is, however, to be noted that beyond exhibiting high-intensity single peaks (so-called singlets) that facilitate their straightforward quantification, each of these classes of metabolite also possesses a biological function that reasonably implicates it in the existing narrative of multiple sclerosis pathology. Perhaps unsurprisingly, then, all three compounds, among others, have previously demonstrated abnormalities relative to control in brain ^1^H-MRS studies of multiple sclerosis.

### N-Acetyl Aspartate (NAA)

N-acetyl aspartate (NAA) is a small molecule synthesized predominantly in mature neurons from acetate and acetyl-coenzyme A. In addition to displaying concentration abnormalities in a number of neurological disorders and injuries, it may serve in part as a storage and transport reservoir for acetate used in myelin lipid anabolism. N-acetyl aspartylglutamate (NAAG) is also predominantly localized to the neurons, though of a smaller range than N-acetyl aspartate, and may modulate the release of neurotransmitters in a variety of pathways (20). The acetyl moieties of both molecules exhibit high-amplitude singlets at 2.01 (N-acetyl aspartate) or 2.04 (N-acetyl aspartylglutamate) ppm; both molecules also exhibit additional multiplets, especially from aspartate in the 2.5–2.7 and 4.4–4.6 ppm range as well as further signals from the amine (N-acetyl aspartate) and glutamate (N-acetyl aspartylglutamate) moieties (21).

Creatine-referenced N-acetyl aspartate has shown reductions relative to control in mixed or unspecified multiple sclerosis lesions (22–35), white matter (36), normal-appearing white matter (22, 24, 25, 30, 34, 37–46), and mixed tissue (47); in relapsing-remitting multiple sclerosis lesions (48–56), white matter (36, 54, 57–64), normal-appearing white matter (48, 50–52, 65–70), gray matter (54, 70), mixed tissue (54, 58, 63, 71–75), and spine (76); and in progressive multiple sclerosis lesions (50, 52, 77–79), white matter (36, 60, 62, 80), normal-appearing white matter (45, 46, 50, 52, 66, 69, 78, 79, 81, 82), gray matter (83), and mixed tissue (71, 73, 84–89). In addition, N-acetyl aspartate quantified as institutional units or relative to non-creatine references like water or phantom acquisitions has been shown to decrease in mixed or unspecified multiple sclerosis lesions (22, 29, 90–96), white matter (97, 98), normal-appearing white matter (22, 91, 94, 99–105), gray matter (94, 100, 103, 104, 106), mixed tissue (107, 108), spine (109–112), and whole-brain measures (113, 114); in relapsing-remitting lesions (50, 53, 96, 115–117), normal-appearing white matter (50, 67, 115, 117–121), gray matter (70, 115, 118, 119, 122), mixed tissue (123, 124), and whole-brain measures (125–128); and in progressive lesions (50, 78, 96, 129), white matter (62, 80), normal-appearing white matter (50, 78, 82, 120, 121, 129–131), gray matter (106, 121, 131–133), mixed tissue (88, 130, 134, 135), spine (136), and whole-brain measures (126).

### Creatine (Cr)

Creatine (Cr) and phosphocreatine (PCr) support the equilibrium of phosphorylated adenosine species useful for cellular energy metabolism by way of kinase enzymes that shuttle phosphates among these molecules (20). Both molecules exhibit overlapping singlets at 3.0 ppm and 3.9 ppm from methyl and methylene, respectively, in addition to amine signals (21). The effect of multiple sclerosis on creatine concentrations in the central nervous system is still unclear, as creatine has been suggested to increase in mixed or unspecified multiple sclerosis lesions (92) and normal-appearing white matter (37); in relapsing-remitting multiple sclerosis lesions (137), normal-appearing white matter (50, 69, 137), and mixed tissue (123); and in progressive multiple sclerosis lesions (50), white matter (62), normal-appearing white matter (50, 69), and mixed tissue (84, 135). It has also demonstrated decreases, however, in unspecified and mixed multiple sclerosis lesions (94), normal-appearing white matter (105), and gray matter (103); in relapsing-remitting lesions (116); and in progressive gray matter (133) and mixed tissue (88).

### Choline (Cho)

Choline-containing compounds, the majority of which are attached to the phospholipid membrane (much of choline or Cho not visible to ^1^H-MRS) but also found as small molecules in aqueous solution (phosphocholine or PCho and glycerophosphocholine or GPC), are thought to be taken into brain tissue through the blood-brain barrier as choline and represent the precursors and byproducts of phospholipid membrane metabolism (20). Mobile choline and phosphocholine exhibit high-intensity methyl singlets at 3.2 ppm with additional methylene multiplets at 4.1–4.3 and 3.5–3.6 ppm, while glycerophosphocholine demonstrates a range of complex resonances from 3.2 to 4.3 ppm (21). Creatine-referenced choline has demonstrated increases in mixed or unspecified multiple sclerosis group lesions (23–25, 34, 41, 138) and normal-appearing white matter (34, 41); in relapsing-remitting lesions (49, 139, 140), normal-appearing white matter (141), and spine (142); and in progressive lesions (78) and spine (76). Decreases, however, have also been shown in mixed multiple sclerosis lesions (29) and in relapsing-remitting lesions (143), normal-appearing white matter (66), gray matter (144), and mixed tissue (144, 145). Increases in choline quantified otherwise have been shown in mixed multiple sclerosis lesions (27, 90, 92) and normal-appearing white matter (92, 101); in relapsing-remitting lesions (137, 146), white matter (64), normal-appearing white matter (137), and gray matter (122); and progressive mixed tissue (84, 135), but decreases have also been demonstrated in mixed multiple sclerosis lesions (91, 94), normal-appearing white matter (105), and gray matter (94); relapsing-remitting lesions (116) and gray matter (119); and progressive mixed tissue (130).

### Inositols (Ins)

Inositols are cyclic organic molecules comprising nine different isomers, of which myoinositol and scylloinositol are the most abundant in human tissue. Myoinositol (mIns) can be found intracellularly within both glial cells and some neuronal types, where it serves as an osmolyte and metabolic precursor to a class of signaling molecules (20). Inositols, particularly myoinositol, have demonstrated central nervous system abnormalities in multiple sclerosis. Creatine-referenced inositol or myoinositol has been shown to increase in mixed or unspecified multiple sclerosis lesions (24, 28, 29, 138, 147); in relapsing-remitting lesions (49, 53, 139, 140, 148), normal-appearing white matter (67), and mixed tissue (84); and in progressive mixed tissue (84) and spine (76), while inositols otherwise quantified have demonstrated increases in multiple sclerosis lesions (29, 92), normal-appearing white matter (92, 99, 101, 102, 110), and gray matter (99, 102, 106); relapsing-remitting multiple sclerosis lesions (53), normal-appearing white matter (67, 69, 117, 119), and mixed tissue (124); and progressive lesions (136), normal-appearing white matter (69, 131), gray matter (106), and mixed tissue (84).

### Glutamate (Glu), Glutamine (Gln), and γ-Aminobutyric Acid (GABA)

Excitatory neurotransmitter glutamate (Glu) and inhibitory neurotransmitter γ-aminobutyric acid (GABA) have also been implicated in multiple sclerosis pathology. As discussed in the section Acquisition Methods, isolating glutamate from its metabolic precursor and spectral neighbor glutamine (Gln) (20) is difficult at magnetic field strengths of 3 T and below, so ^1^H-MRS experiments involving this metabolite in multiple sclerosis have been sparse. Glutamate concentration not referenced to creatine has shown decreases in multiple sclerosis mixed tissue (107) and relapsing-remitting mixed tissue (149) but also increases in multiple sclerosis lesions (92) and normal-appearing white matter (92, 100).

GABA is naturally present at comparatively low concentrations in the brain, and the signal amplitude of its peaks is further reduced by *J*-coupling interactions among its protons. Isolating its signature from those of nearby metabolites choline and creatine is therefore difficult in one-dimensional spectroscopy without additional spectral editing methods. While research on the molecule is therefore limited, edited GABA concentration has been shown to decrease in relapsing-remitting (150) and progressive (134) mixed tissue.

### Glutathione (GSH)

Limited work using ^1^H-MRS has also been performed to assess the effects glutathione (GSH) in the brain. Like GABA, glutathione possesses a low-amplitude spectral signature that overlaps heavily with metabolites of much higher brain concentrations, therefore requiring spectral editing for accurate quantification by one-dimensional ^1^H-MRS. Previous research employing this editing has suggested that multiple sclerosis is associated with glutathione decreases in gray but not white matter voxels measured superior to the ventricles (104); some evidence also exists of reduced glutathione concentration in secondary progressive mixed-tissue voxels in the frontal (151, 152) and parietal (152) cortex.

### Lactate (Lac)

Lactate is a byproduct of pyruvate reduction in anaerobic glycolysis that is thought to serve as an alternative cellular energy source during high levels of neural activity. While transient increases of lactate have been observed in healthy brain, its prolonged presence in neural tissue is typically considered a mark of pathology (20). Accordingly, some evidence exists of enhanced lactate signal in mixed or unspecified multiple sclerosis lesions (24, 31, 153) and relapsing-remitting lesions (140).

## Study Cohort Demographics

### Multiple Sclerosis Phenotypes

While a number of studies have employed experiment groups of undefined (30–32, 47, 93, 104, 138, 147, 153–157) or mixed (22–28, 33, 34, 36–38, 40–43, 81, 90–92, 94, 97, 100–103, 105, 107–111, 113, 114, 158–168) multiple sclerosis phenotypes, a significant body of literature focuses on the metabolic underpinnings of distinctly relapsing-remitting, secondary progressive, and primary progressive multiple sclerosis variants.

Relapsing-remitting multiple sclerosis (RR-MS) is marked by months to years of clinical quiescence punctuated by subacute neurological relapses of paresthesia, paresis, loss of balance and coordination, anopsia, dysautonomia, cognitive dysfunction, and other symptoms. These clinical manifestations are usually accompanied by the presence of hyperintense white matter lesions on *T*_2_-weighted MRI or gadolinium-enhancing lesions evident in *T*_1_-weighted MR sequences (169). Gadolinium-enhancing lesions lose contrast enhancement over the course of several weeks (170) but may remain as hyperintensities on *T*_2_-weighted images, some of which also manifest as usually irreversible hypointensities on *T*_1_-weighted images suggestive of inflammatory edema, demyelination, and, when chronic, axonal loss (171). ^1^H-MRS in gadolinium-enhancing acute lesions of relapsing-remitting multiple sclerosis has demonstrated lower concentrations of N-acetyl aspartate, creatine, and glutamate-glutamine with increases in choline and myoinositol (116) and sometimes increased lactate (140).

Progressive multiple sclerosis (P-MS), by contrast, exhibits a relative absence of clinical relapses and comparatively less focal inflammatory central nervous activity, manifesting instead as functional deterioration with neurodegeneration, marked by diffuse white matter injury and greater rates of cortical atrophy. Most cases of progressive multiple sclerosis are secondary progressive (SP-MS) transitions from a relapsing disease course. A minority of patients develop a progressive form from the outset, a disease course which is then termed primary progressive multiple sclerosis (PP-MS) (169).

**Figure 1:**
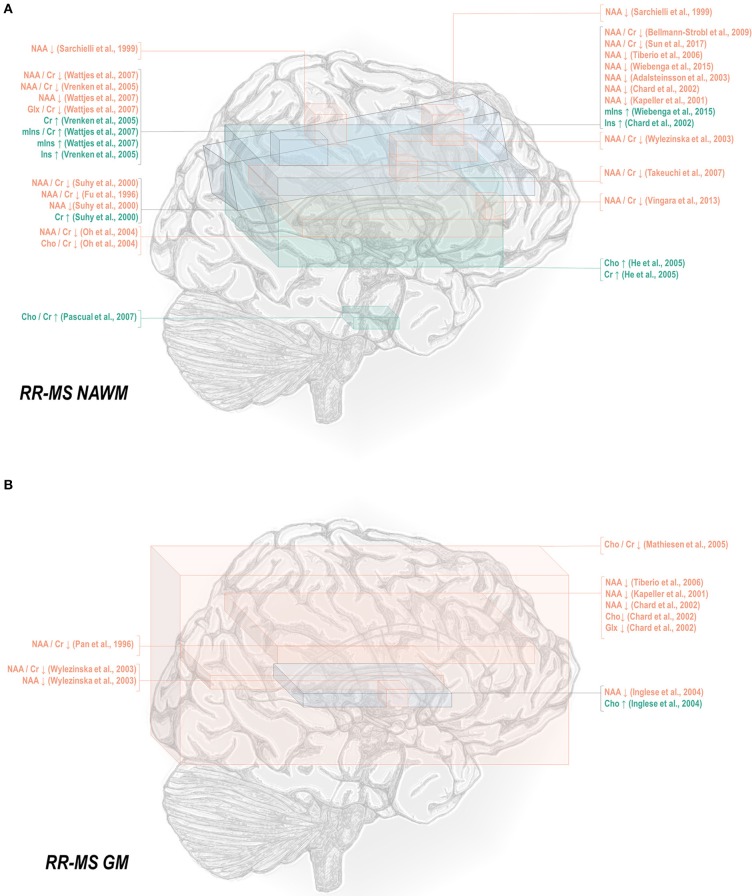
Relapsing-remitting multiple sclerosis is associated with a number of metabolic changes as measured by proton magnetic resonance spectroscopy in both white and gray matter. **(A)** Previous applications of proton magnetic resonance spectroscopy (^1^H-MRS) in the normal-appearing white matter (NAWM) of relapsing-remitting multiple sclerosis (RR-MS) patients have demonstrated decreases in N-acetyl aspartate (NAA) and glutamate-glutamine (Glx), increases in creatine (Cr), and inositols (Ins), and either decreases or increases in choline (Cho) relative to control. **(B)** Similar analyses in gray matter (GM) have demonstrated decreases in N-acetyl aspartate and glutamate-glutamine with either decreases or increases in choline.

**Figure 2:**
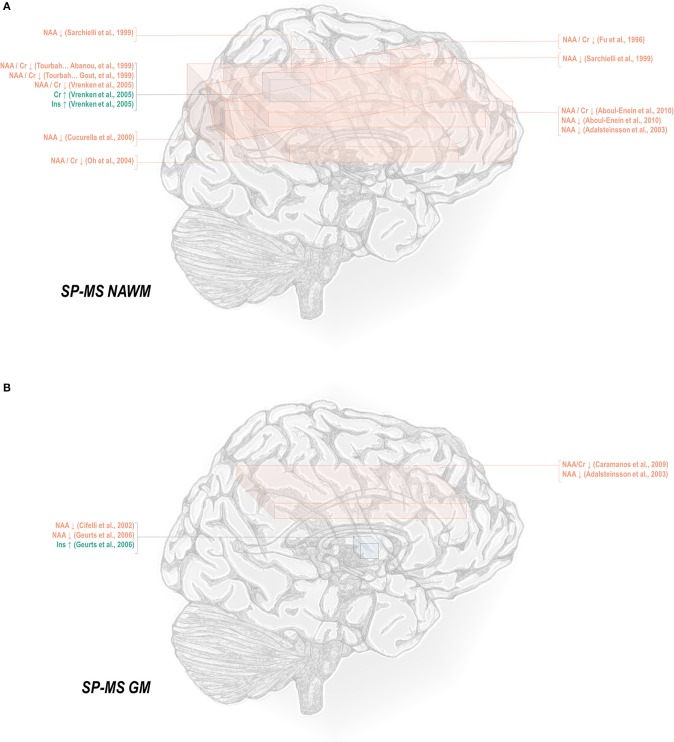
Secondary progressive multiple sclerosis is associated with a number of metabolic changes as measured by proton magnetic resonance spectroscopy in both white and gray matter. **(A)** Previous applications of proton magnetic resonance spectroscopy (^1^H-MRS) in the normal-appearing white matter (NAWM) of secondary progressive multiple sclerosis (SP-MS) patients have demonstrated decreases in N-acetyl aspartate (NAA) with increases in creatine (Cr) and inositols (Ins). **(B)** Similar analyses in gray matter (GM) have demonstrated decreases in NAA with increases in inositols.

Normal-appearing white matter has demonstrated similar metabolic signatures in both conditions. Relapsing-remitting normal-appearing white matter has demonstrated reductions in N-acetyl aspartate normalized to creatine (48, 50–52, 65–70), choline (67), or other references (50, 67, 115, 117–121), and lower creatine-referenced choline (66) as well as higher creatine (50, 69, 137), creatine-referenced myoinositol (67), and inositol (67, 69, 117, 119) than controls (Figure 1A). Secondary progressive normal-appearing white matter has similarly demonstrated lower N-acetyl aspartate normalized to creatine (46, 52, 66, 69, 79), choline (78), or other references (78, 120, 121); and higher creatine (69) and inositols (69) than controls (Figure 2A).

In normal-appearing white matter of relapsing-remitting multiple sclerosis only, additional findings of lower creatine-referenced glutamate-glutamine (67), increases in creatine-referenced choline (141), and an increase in choline (137) have also been reported. It is uncertain from these reports alone whether the finding of these metabolic abnormalities in relapsing-remitting but not secondary progressive multiple sclerosis reflects not true metabolic differences in the two diseases but rather lack of research. A multitude of spectroscopic analyses on the normal-appearing white matter of relapsing-remitting multiple sclerosis have reported null results in either metabolite, and while several have similarly addressed normal-appearing white matter choline (46, 66, 69, 78–80, 120, 172) in secondary progressive multiple sclerosis, only a handful have done so for glutamate or glutamine (69, 172, 173). One rare study reporting group statistics for both relapsing-remitting and secondary progressive multiple sclerosis normal-appearing white matter demonstrated an aggregate increase in glutamate regardless of phenotype (100). The limited number of studies directly comparing both subtypes have additionally demonstrated greater abnormality in secondary progressive than relapsing-remitting multiple sclerosis, with larger decreases in N-acetyl aspartate referenced to creatine (36, 79, 80) and otherwise (80, 105, 120) in areas of white matter, a difference mirrored in research in which normal-appearing white matter in central brain (66) and centrum semiovale (79) demonstrated differences from control in N-acetyl aspartate in the secondary progressive but not the relapsing-remitting phenotype. Similarly, one study showed increases from control in creatine in white matter for secondary progressive but not relapsing-remitting multiple sclerosis (62), though the region of interest under investigation notably contained lesions in all disease groups.

A smaller number of studies have examined the effects of each subtype on gray matter metabolism. Relapsing-remitting gray matter has demonstrated decreases of N-acetyl aspartate referenced to creatine (54, 70) and other metrics (70, 115, 118, 119, 122) (Figure 1B). Similarly, secondary progressive gray matter has demonstrated decreases in N-acetyl aspartate referenced to creatine (83) or otherwise (106, 121, 132) (Figure 2B). The literature on secondary progression has not, however, replicated either decreases (119, 144) or increases (122) in choline or decreases in glutamate-glutamine (119) found in relapsing-remitting gray matter, and, unlike the literature on gray matter in relapsing-remitting multiple sclerosis, has demonstrated increases in inositol (106) relative to controls. Published spectroscopic analyses comparing gray matter specifically in relapsing-remitting and secondary progressive multiple sclerosis are sparse, but, similarly to the literature on normal-appearing white matter, have demonstrated decreases in N-acetyl aspartate in secondary progressive but not relapsing-remitting multiple sclerosis (83, 121), in addition to the aforementioned increases in inositol in secondary progressive but not relapsing-remitting multiple sclerosis (106).

Mixed-tissue voxels typically contain a significant proportion of gray matter; studies thereof may thus be queried to supplement understanding of metabolic similarities and differences between relapsing-remitting and secondary progressive gray matter. Mixed-tissue voxels in relapsing-remitting multiple sclerosis have demonstrated decreased N-acetyl aspartate referenced to creatine (54, 58, 63, 71–74) or otherwise (123, 124); increased creatine (123); increased inositol referenced to creatine (84) or otherwise (124); and decreased GABA plus homocarnosine and macromolecules, called GABA+ (150). Similarly, studies on mixed-tissue voxels in secondary progressive multiple sclerosis have reported decreases in N-acetyl aspartate referenced to creatine (71, 73, 84, 85, 88) and otherwise (88, 134, 135), as well as increases in creatine (84, 135) and inositol referenced to creatine or otherwise (84), and decreases in GABA (134) but have not replicated relapsing-remitting findings of decreased creatine-referenced choline (144, 145), glutamate (149), or glutamate-glutamine (124, 149). Secondary progressive but not relapsing-remitting patients have, however, exhibited decreases in mixed-tissue creatine (88) and glutathione (151, 152) as well as increases in choline (135). The handful of analyses concomitantly examining mixed-tissue voxels in both phenotypes have reported decreases in N-acetyl aspartate in either both patient groups (71) or secondary progressive multiple sclerosis only (84, 85, 88) and increases in creatine-referenced inositol in both patient groups, in addition to increases in inositol and creatine in secondary progressive but not relapsing-remitting multiple sclerosis (84).

**Figure 3:**
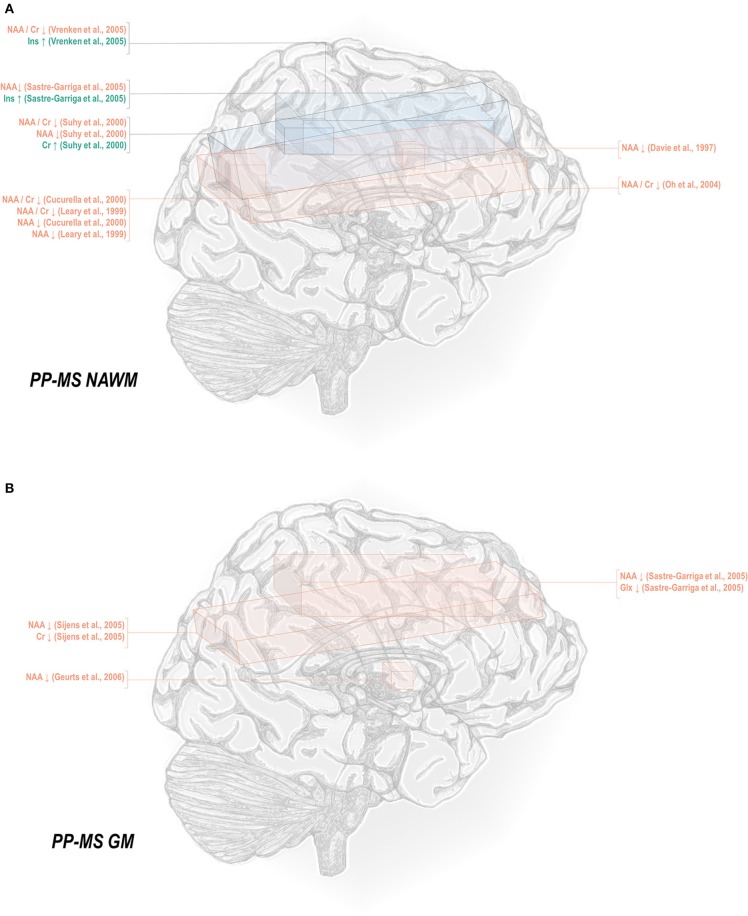
Primary progressive multiple sclerosis is associated with a number of metabolic changes as measured by proton magnetic resonance spectroscopy in both white and gray matter. **(A)** Previous applications of proton magnetic resonance spectroscopy (^1^H-MRS) in the normal-appearing white matter (NAWM) of primary progressive multiple sclerosis (PP-MS) patients have demonstrated decreases in N-acetyl aspartate (NAA) with increases in creatine (Cr) and inositols (Ins). **(B)** Similar analyses in gray matter (GM) have demonstrated decreases in NAA, creatine, and glutamate-glutamine (Glx).

Less thoroughly studied in the spectroscopy literature is the primary progressive phenotype, which comprises only about 15% of all cases. Primary progressive multiple sclerosis normal-appearing white matter has demonstrated decreases in N-acetyl aspartate referenced to creatine (45, 50, 69, 78, 82), choline (78), and otherwise (50, 78, 82, 129, 131), as well as increases in inositol (69, 131) and creatine (50) (Figure 3A). Primary progressive gray matter has similarly demonstrated decreases in N-acetyl aspartate (106, 131, 133) but also in creatine (133) and glutamate-glutamine (131) (Figure 3B).

A handful of papers makes reference to a relapsing-progressive multiple sclerosis patient case (86) or group (174), described in the latter as “in a progressive phase of the disease after having shown remissions” and therefore ambiguous in its distinction from the secondary progressive phenotype. Alternatively, some studies include progressive-relapsing patients within mixed-subtype cohorts (101, 113, 163, 164). It bears emphasis, however, that current recommendations suggest that “relapsing-progressive” lacks a consensus definition and therefore not be reified as a phenotype distinct from that of secondary progressive disease (175); and that “progressive-relapsing” be re-categorized as “primary progressive with disease activity” (176).

A few studies include experimental groups defined by “progressive multiple sclerosis” (81, 130, 177), including patients with either the primary or secondary progressive variant. Both phenotypes exhibit similar patterns of clinical decline, albeit in primary progressive without a preceding relapse-onset phase, as well as poorer response to disease-modifying therapies that can have striking therapeutic effects in relapsing-remitting cases (178). The existence of some treatment-responsive subgroups in drug trials for progressive multiple sclerosis has indicated, however, that the distinction not only between relapsing and progressive disease phenotypes but also among different progressive patients may lie in the relative contributions of shared disease mechanisms rather than qualitative differences among the mechanisms themselves (178). It may be argued that primary progressive multiple sclerosis exhibits the greatest contribution from mechanisms independent of autoimmunity, as indicated by the lower proportion of women, who typically exhibit higher incidence of autoimmune diseases than men, expressing this phenotype relative to the others (179). This possibility may caution against the conflation of primary with secondary progressive multiple sclerosis in the continued search for diagnostic biomarkers measurable by ^1^H-MRS.

Finally, some *in vivo* proton spectroscopy work has examined the metabolic signatures of clinically isolated syndrome (CIS) (67, 180–183) and radiologically isolated syndrome (RIS) (184, 185), including to predict conversion of individuals with these often prodromal syndromes to clinically definite multiple sclerosis. A comprehensive or detailed treatment of the existing literature thereof stands outside the scope of the present review, which centers instead on studies of the disease phenotypes defined above. Its relative sparsity, however, particularly marked for radiologically isolated conditions, highlights a potentially fruitful avenue for future investigation into disease evolution from the earliest stages of imaging and symptomatic manifestation.

### Age and Disease Duration

A number of studies within the multiple sclerosis literature have examined and found no significant correlations in controls between age and mixed tissue N-acetyl aspartate, creatine, choline, or inositol (84); or in multiple sclerosis patients between age and concentrations of whole-brain N-acetyl aspartate (186, 187); creatine-referenced N-acetyl aspartate in white matter (36); or creatine-referenced N-acetyl aspartate, choline, or myoinositol in chronic or acute lesions or normal-appearing white matter (148) or in parietal gray or white matter (63); similarly, no relationship was found between age and concentrations of creatine-referenced N-acetyl aspartate, choline, myoinositol, or macromolecules, or the magnitude of the creatine signal itself, in normal-appearing white matter or non-enhancing lesions (48). Other research has reported in control white matter significant positive relationships between age and concentrations of creatine (50, 101) and myoinositol (101), neither metric replicated in multiple sclerosis patients (50, 101), in addition to a significant inverse relationship in multiple sclerosis patients between age and whole-brain N-acetyl aspartate (127), spinal N-acetyl aspartate (29), creatine-referenced N-acetyl aspartate in gray or white matter (188), and normal-appearing white-matter glutamate-glutamine (131). Finally, concentrations of white matter and thalamic myoinositol, white matter creatine, and white matter and thalamic choline (99), in addition to the magnitude of the unsuppressed signal from water in white matter (48), have all been shown to increase with age.

**Figure 4:**
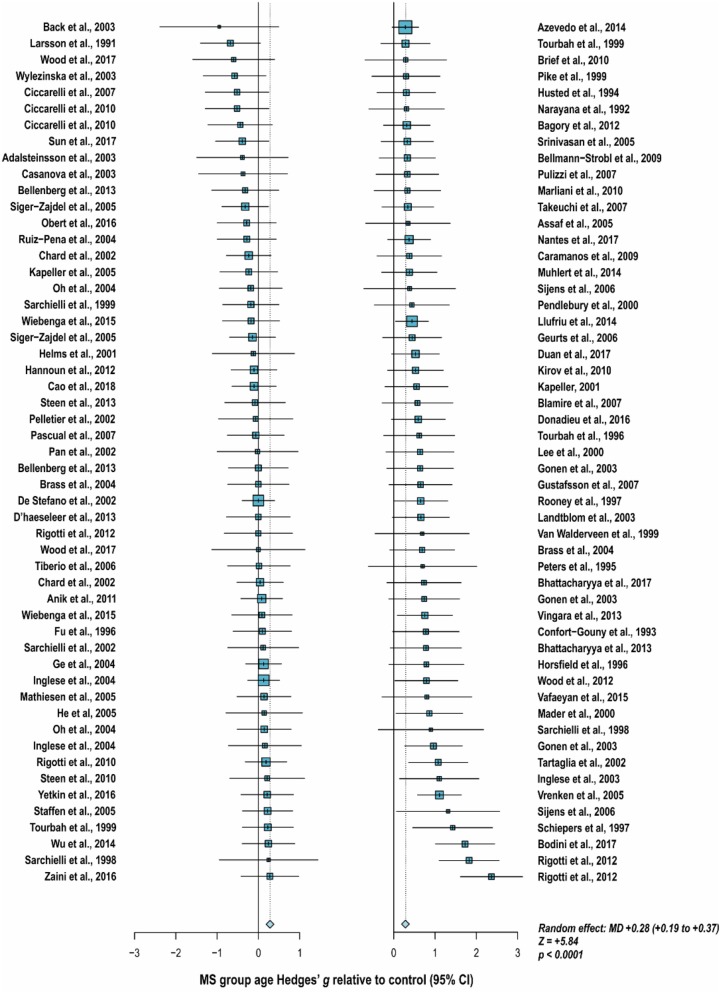
Cross-sectional analyses of relapsing-remitting and mixed multiple sclerosis subtypes published between 1991 and 2018 have reported comparing patient groups with age-matched or younger control groups. Shown are the standardized mean differences between MS patient and control groups of 106 ^1^H-MRS studies published between 1991 and 2018 that report means as well as standard deviations and/or range for group ages examining metabolic differences in the brain and/or spinal cord between individuals with and without relapsing-remitting, unspecified, or mixed subtypes of multiple sclerosis. A random-effects model exhibited a significant effect of group on subject age across the 106 comparisons, with an overall Hedges' *g* of +0.28 ± 0.09 (mean difference +2.5 ± 0.8 years) and significance level of *p* < 0.0001. Many analyses attempted to compensate for such age differences in their statistical modeling procedures. Marker area is weighted by group size. MS: multiple sclerosis; MD: standardized mean difference, reported as Hedges' *g*; CI: confidence interval.

**Figure 5:**
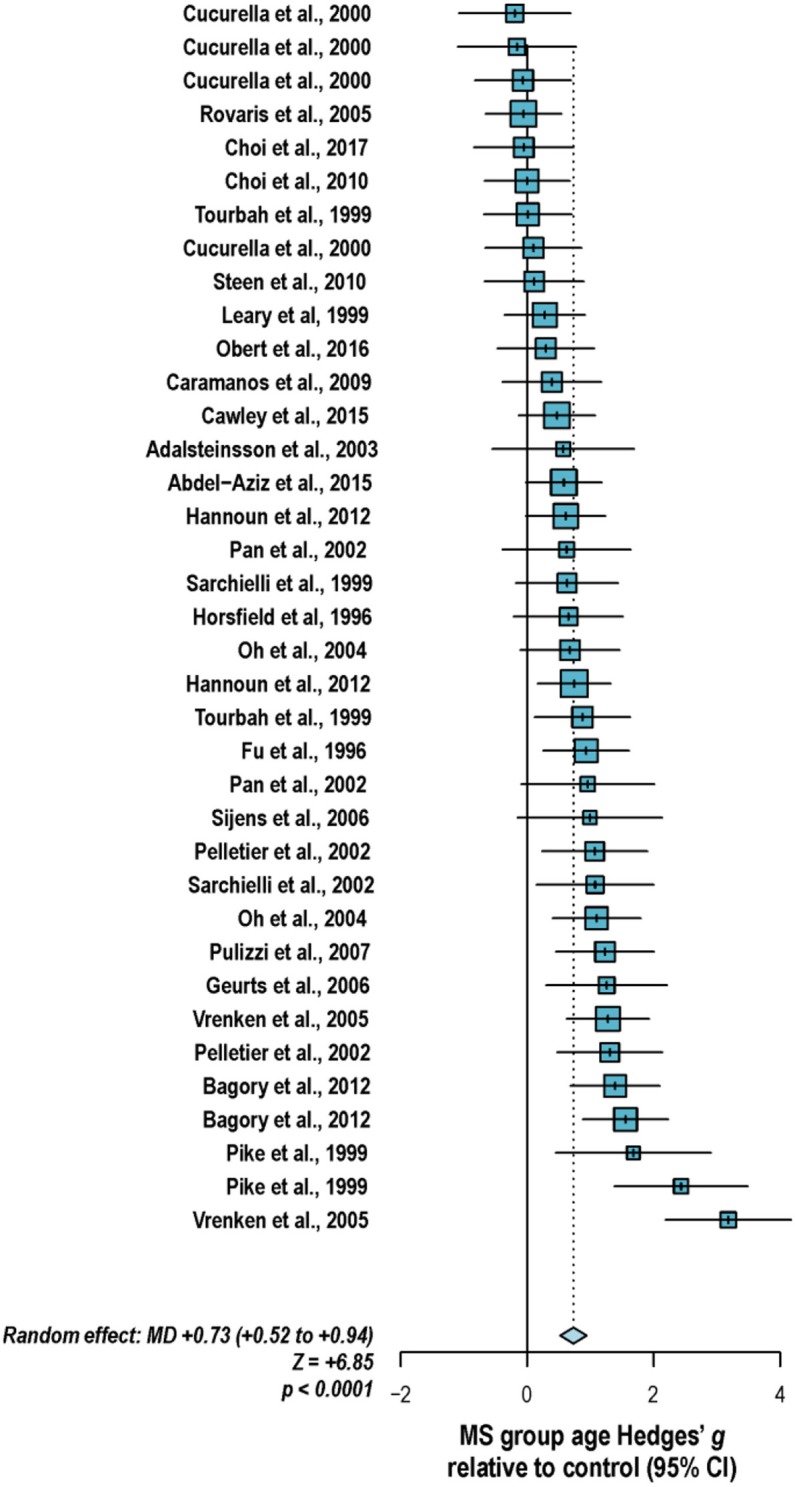
Cross-sectional analyses of progressive multiple sclerosis published between 1996 and 2017 have reported comparing patient groups with age-matched or substantially younger control groups. Shown are the mean differences between multiple sclerosis patient and control groups of 37 ^1^H-MRS studies published between 1996 and 2017 that report means as well as standard deviations and/or range for group ages examining metabolic differences in the brain and/or spinal cord between individuals with and without primary progressive, secondary progressive, progressive relapsing or chronic progressive, or mixed progressive subtypes of multiple sclerosis. A random-effects model exhibited a significant effect of group on subject age across the 37 comparisons, with an overall Hedges' *g* of +0.73 ± 0.21 (mean difference +6.7 ± 1.9 years) and significance level of *p* < 0.0001. Many analyses attempted to compensate for such age differences in their statistical modeling procedures. Marker area weighted by group size. MS: multiple sclerosis; MD: standardized mean difference, reported as Hedges' *g*; CI: confidence interval.

With these potentialities under consideration, a large proportion of cross-sectional spectroscopic analyses on multiple sclerosis have included explicit age matching between disease groups and control in their study designs. These endeavors have been marked by varying levels of success. A random-effects model (189) of 106 cross-sectional comparisons of multiple healthy controls and patients with relapsing-remitting multiple sclerosis, mixed multiple sclerosis subtypes, or multiple sclerosis of no designated subtype published from 1991 to 2018, using a previously reported method for interpolating standard deviations (190) in 32 cases, found a mean difference of +2.5 ± 0.8 years in patient relative to control ages, corresponding with a Hedge's *g* of 0.28 ± 0.09 (*p* < 0.0001; Figure 4). A similar analysis of 37 cross-sectional investigations between controls and patients with progressive multiple sclerosis (including progressive, primary progressive, and secondary progressive, as well as “relapsing-progressive” and “chronic progressive”) published between 1996 and 2017, using interpolated standard deviations in 7 cases, found a mean difference of +6.7 ± 1.9 years in patient relative to control ages, corresponding with a Hedge's *g* of 0.73 ± 0.21 (*p* < 0.0001; Figure 5).

What element of the subtype-specific findings currently published, particularly those involving progressive groups, is influenced by differences in age therefore remains a valid question. While this issue can be skirted by exact age-matching among experimental groups (152), some researchers have contended with ineluctable disparities in group demographics by either confirming the lack of a statistically significant correlation between participant age and salient experimental endpoints, as reported above, or including age values in their statistical models [as in, for example, (66)].

Disease duration is another potential confound that may affect multiple sclerosis subtype-specific trends in metabolite ratios and concentrations. Like age, some research has investigated and found no significant correlation between disease duration and whole-brain N-acetyl aspartate (186, 187); cervical spinal N-acetyl aspartate (110); lesion creatine-referenced N-acetyl aspartate (34, 148), choline (34, 148), or myoinositol (148); white matter creatine-referenced N-acetyl aspartate (34, 63, 148), choline (34, 63, 148), or myoinositol (63, 148); creatine-referenced N-acetyl aspartate, choline, or myoinositol in posterior cingulate gyrus (63); N-acetyl aspartate, choline, or creatine in cortical white matter (64) or occipito-parietal cortex (88); N-acetyl aspartate or choline in cerebellar white matter (98); or N-acetyl aspartate, choline, creatine, or myoinositol in central brain (191). Other research has shown that disease duration may vary inversely with whole-brain N-acetyl aspartate (126, 192, 193), white matter N-acetyl aspartate (110, 194), thalamic N-acetyl aspartate (70), and lesion creatine-referenced N-acetyl aspartate (52) as well as directly with normal-appearing white matter creatine (69, 131). As in considerations of group age-matching, it is therefore important to control for the possibility that apparent metabolic differences ostensibly traceable to disease variant or even age are not being driven by this confound either, and vice-versa.

### Sex

Despite the fact that multiple sclerosis, with the possible exception of the primary progressive variant, exhibits a higher and rising predominance in women relative to men (195), few papers have explicitly addressed the question of sex differences in the brain ^1^H-MRS signatures of the disease. These previous reports have found no sex differences in whole-brain N-acetyl aspartate (186); white matter creatine-referenced N-acetyl aspartate (36); or spinal cord creatine, N-acetyl aspartate, or choline (29). Other non-spectroscopic magnetic resonance studies of multiple sclerosis have found sex differences, however, in contrast-enhancing lesion load (196–198), primary progressive *T*_1_-weighted lesion volume (199), and degree of gray matter atrophy (200, 201). To our knowledge no ^1^H-MRS research to date has explicitly examined the potential interactions between sex and disease state on the metabolic signatures of central nervous tissue.

**Figure 6:**
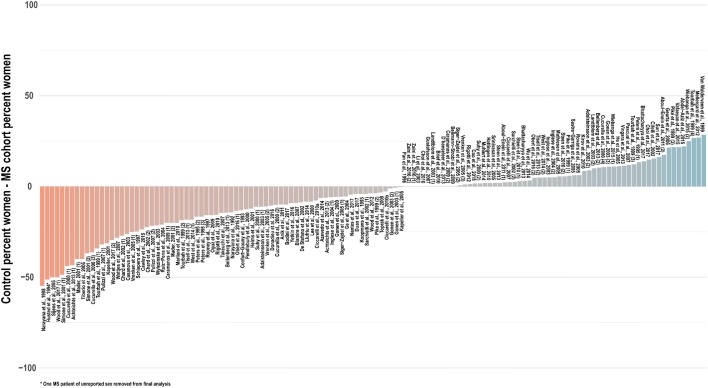
Cross-sectional analyses of relapsing, progressive, unspecified, and mixed subtypes of multiple sclerosis published between 1992 and 2018 have tended to feature control groups with a lower proportion of women than the patient groups with whom they are compared. An investigation of 134 cross-sectional analyses published between 1992 and 2018 that reported group sex ratios found that, on average, control groups contained 6 percentage points fewer women (range 55% fewer to 30% more) than experimentally compared groups of multiple sclerosis patients, with significant disparities from experimental groups in control for both proportion (*t* = −2.9, two-tailed *p* = 0.004) and absolute number (*t* = −2.6, *p* = 0.01) of tested women. Distinct analyses from the same publications denoted in parentheses. MS: multiple sclerosis.

Especially in light of these reported sex differences on imaging scans, in the absence of robust spectroscopic findings to the contrary, it is prudent to assume a potential for sex differences in brain metabolite concentrations and therefore match experiment groups not only for age but also for sex. In only a few studies (35, 42, 54, 57, 65, 83, 105, 152, 202) was the reported sex composition identical between the control and at least one experimental group; in a handful of others, the statistical influence of imperfect sex-matching was assessed by chi-square analysis (51, 56, 107, 203, 204). An investigation of 134 cross-sectional analyses published between 1992 and 2018 that reported group sex ratios found that, on average, control groups contained 6 percentage points fewer women (range 55% fewer to 30% more) than experimentally compared groups of multiple sclerosis patients, with significant disparities from experimental groups in control for both proportion (*t* = −2.9, two-tailed *p* = 0.004) and absolute number (*t* = −2.6, *p* = 0.01) of tested women (Figure 6). As in age-biased analysis, a few groups have attempted to control for this potential confound by adjusting for sex in statistical analysis of disease group effect size (56, 64, 100, 103, 118, 119, 124, 134, 136, 191, 205, 206) or, as mentioned, checking separately for associations between sex and ^1^H-MRS outcomes (29, 36, 103, 186).

## Spectral Acquisition and Processing

### Acquisition Methods

Most research on the brain ^1^H-MRS signatures of multiple sclerosis has been conducted between 1.5 T and 3 T, with only a handful of studies completed at higher field strengths, including 4 T (54, 135, 162, 188, 192, 207, 208) and 7 T (38, 104). ^1^H-MRS data exhibit a trend to higher signal to noise with static field strength due to enhanced spin polarization, in addition to higher spectral dispersion deriving from increased precession frequency. Notably, however, due in part to the influences of spatial inhomogeneity in static (B_0_) and radiofrequency (B_1_) field profile, increased field strength does not guarantee improved spectral quality (209).

The dearth of studies at fields >3 T may be particularly damning for research on multiple sclerosis physiology due to the difficulty of reliably measuring glutamate at low field. Histology research has previously implicated this molecule in key disease processes, suggesting that synaptic glutamate clearance is compromised in multiple sclerosis white matter (210) and that several molecular contributors to glutamate homeostasis are altered in multiple sclerosis lesions (211). Our ability to examine the role of glutamate *in vivo* by brain ^1^H-MRS is limited, however, by the probability that its major resonances from 2.04 to 2.35 ppm can be quantified independently of its metabolic partner glutamine, its own principal multiplets from 2.11 to 2.45 ppm (212). While some sequences may be optimized for the measurement of glutamate at 3 T (213, 214), quantification accuracy for this metabolite has been shown to increase at higher field (215).

As a result, the majority of ^1^H-MRS research examining the role of glutamate in multiple sclerosis has reported values of glutamate conflated with glutamine as glutamate-glutamine or “Glx,” a classification that has yielded a number of null between-group findings in mixed or relapsing-remitting multiple sclerosis thalamus (99, 149), hippocampus (149), acute (216, 217) and chronic (216) lesions, non-enhancing lesions (172), normal-appearing gray (115) and white (115, 172, 217) matter, and *T*_1_-isointense lesions (115), as well as in secondary progressive hippocampus (134), sensorimotor cortex (134), prefrontal cortex (134), normal-appearing white matter (172), and nonenhancing lesions (172) and primary progressive normal-appearing white matter (131). While null results have similarly been yielded in some regions and conditions by studies attempting to quantify glutamate separately from glutamine (69, 92, 106, 117, 149), at least one study has demonstrated alterations in glutamate but not glutamine in the normal-appearing white matter and enhancing lesions (92) of multiple sclerosis patients, suggesting that null findings in Glx could sometimes be influenced by variable but ultimately unremarkable glutamine levels obfuscating potentially systematic abnormalities in glutamate.

In addition to acquisition at higher field strengths to maximize spin polarization and spectral dispersion, studies optimized to quantify metabolites with fast-decaying multiplet resonances like glutamate and the inositols should be conducted at as low an echo time as possible to optimize the signal to noise ratio of these species' already low and broad spectral signatures. Among 197 publications surveyed from 1990 to 2018 that reported the use of ^1^H-MRS to investigate the brain or spinal tissue in at least one individual with multiple sclerosis, over 45% included sequences of echo times (*T*_*E*_) <40 ms. Among these, at least 26 publications (23, 24, 27, 35, 46, 53, 69, 77, 79, 90–93, 95, 106, 116, 138, 139, 147, 153, 154, 160, 172, 216–219) employed Stimulated Echo Acquisition Mode (STEAM) localization instead of the more commonly used Point-RESolved Spectroscopy (PRESS), of which the former facilitates lower echo times and enables a sharper volume profile at the expense of 50% signal loss relative to the latter. Other short- and long-*T*_*E*_ spectral acquisition sequences used have included PRESS with spectral editing (MEscher-GArwood Point-RESolved Spectroscopy or MEGA-PRESS) for GABA (107, 134, 150), chemical shift imaging (CSI) with multiple quantum filtering (151, 152) or band-selective inversion (104) for glutathione, and diffusion-weighted PRESS (38, 103, 206) or STEAM (220), though the latter was used to probe diffusion properties rather than metabolite concentrations.

Almost half of the surveyed papers employed magnetic resonance spectroscopic imaging (MRSI) instead of single-voxel acquisition schemes, including echo-planar spectroscopic imaging (EPSI) (85, 89, 124, 144, 145, 221–223), and other CSI or spectroscopic imaging sequences encoded in two (25, 28, 30, 34, 36, 41, 43, 45, 50, 52, 54, 57, 60–62, 64–66, 68, 72, 73, 80, 81, 83, 87, 93, 94, 115, 119, 130, 131, 133, 135, 141, 159, 162–165, 167, 168, 174, 188, 191, 194, 204, 224–231) or three (102, 121, 122, 137, 202, 232, 233) spatial dimensions. MRSI offers the obvious advantage of enabling metabolic profiling over a large area of multiple tissue types, thereby enabling averaging over gray matter, white matter, and lesions within the same individual, enabling more robust estimates for the metabolic patterns of hypothetically pure tissue (102, 104, 163, 164). The ability to compare spectral outputs against compositional parameters of multi-voxel scans has also been exploited to calculate the water proton signal inherent in a hypothetical cerebrospinal fluid (CSF) voxel by regressing over the CSF partial volumes of multiple MRSI voxels to use the calculated water signal as an internal metabolite quantification reference (162). In addition, MRSI facilitates the investigation of possible regional disparities in disease-related abnormality, independent of lesion localization. A number of papers examining multiple brain regions have found multiple sclerosis to be associated with distinct metabolic abnormalities in different areas of the brain, among, for example, mixed voxels in posterior cingulate cortex, medial PFC, and left hippocampus (150); mixed voxels in sensorimotor cortex, prefrontal cortex, and hippocampus (134); cortical gray matter, thalamus, and hippocampus (106); and mixed voxels in cingulate cortex, parietal cortex, thalamus, and hippocampus (149).

### Spectral Quality

The quality of a spectrum obtained by a ^1^H-MRS experiment is measured predominantly by two related metrics: full width at half maximum (FWHM) of singlet resonances (generally N-acetyl aspartate, creatine, and choline) and signal to noise ratio (SNR) measured from the same peaks.

Within the context of *in vivo* ^1^H-MRS, FWHM describes the frequency bandwidth covered at half-height by the singlet used to calculate it. Because a spectrum in the frequency domain represents the Fourier transform of the free induction decay (FID) measured in time domain from excited precessing spins, resonances represented by Lorentzian singlets in frequency domain reflect the exponential decay dynamics of their associated signals in time domain. In particular, the FWHM of a frequency-domain singlet increases with the rate at which its corresponding time-domain FID decays as a result of *T*_2_^*^, driven not only by chemical properties of the nucleus at hand but also by the degree of dephasing imposed by static field inhomogeneity from spatial disparities in the magnetic susceptibilities of local tissues.

SNR describes the amplitude of one or more singlet resonances relative to the standard deviation of peak amplitudes measured over the spectral noise floor; in proton spectroscopy measures this noise denominator is conventionally doubled (234). Its value depends on a number of factors, including the proton density of the species in question, the size of the measured voxel, the receive bandwidth for spectral acquisition, and the flip angle due to the effective B_1_ fields imposed by RF transmission. Because increasing the width of a spectral peak will reduce its amplitude at constant area, increased FWHM is associated with lower SNR for a metabolite of fixed concentration.

Spectral quality as measured by both FWHM and SNR has been shown to influence the apparent concentrations of metabolites as quantified by ^1^H-MRS. Simulations of metabolite spectra at 1.5 T, for example, have associated decreases in SNR with significant increases in the standard deviations of quantified concentrations in five commonly quantified metabolites (N-acetyl aspartate, creatine, choline, myoinositol, and glutamate-glutamine) (235). Because of spectral dispersion differences among measurements taken at different field strengths, these patterns may not be generalizable to those at higher static fields. Simulation experiments representing short-*T*_*E*_ experiments in the human hippocampus at 4 T have, however, similarly shown that apparent concentration in at least thirteen metabolites is sensitive to both FWHM and especially SNR, with variable influence on each metabolite (236); simulations of both short-*T*_*E*_ and editing experiments for GABA and glutathione at 7 T have also demonstrated metabolite-specific relationships between quantification error variance and these spectral quality parameters (237).

Few papers report group comparisons of FWHM in multiple sclerosis vs. control individuals. Water FWHM has been found to be similar between relapsing-remitting patients and controls in posterior cingulate cortex, medial prefrontal cortex, and left hippocampus (150). Within relapsing-remitting patients, metabolite spectral FWHM has also been found to be comparable across chronic lesions, normal-appearing white matter, and cortical gray matter (224). Other research, however, has noted increases in aggregate N-acetyl aspartate, creatine, and choline singlet FWHM in secondary progressive relative to relapsing-remitting patients in cortical gray matter as well as that of primary progressive patients in the thalamus, with values comparable across relapsing-remitting, secondary progressive, and primary progressive multiple sclerosis and control in the hippocampus as well as between multiple sclerosis patients taken together and control in all three regions (106). By contrast, significant differences in metabolite singlet FWHM were not reported among relapsing-remitting multiple sclerosis, secondary progressive multiple sclerosis, and control in normal-appearing white matter or white-matter lesions (172), or among relapsing-remitting, secondary progressive, and primary progressive patients and control in normal-appearing white matter (69).

Decreased SNR has been noted in relapsing-remitting and primary progressive patients relative to control in thalamus or hippocampus but not cortical gray matter, reflective of decreases in the amplitude of the N-acetyl aspartate singlet in multiple sclerosis groups (106). SNR has also been observed to be lower in an edematous lesion than in a comparable region of healthy white matter, though this difference may have been attributable to the smaller measured volume of the former (116). This interpretation is supported by findings of comparable SNR in equal volumes within chronic lesions, normal-appearing white matter, and cortical gray matter in multiple sclerosis patients (224) and in normal-appearing white matter among individuals with relapsing-remitting, secondary progressive, and no multiple sclerosis (172) or relapsing-remitting, primary progressive, secondary progressive, or no multiple sclerosis (69).

Notably, SNR decreases with diffusion due to signal dephasing in spins flowing parallel with the spatial axis of variation in a pair of otherwise balanced gradients. Exploited by diffusion-weighted imaging sequences to locate regions of abnormal tissue microstructure, this property may reduce SNR in voxels in which measured spins exhibit abnormally high diffusivity.

Finally, differential proportions of non-tissue relative to tissue water within a measured voxel, as in a previous work demonstrating higher partial volumes of CSF than control in a frontal cortex voxel from patients with progressive multiple sclerosis (238), may also affect its spectral quality when shim routines are optimized to water signal, which encompasses both CSF and tissue compartments, while metabolites are typically limited to areas of tissue only.

### Macromolecule and Lipid Contributions to Spectroscopic Baseline

Complicating but also potentially complementing the measurement of small-molecule metabolites traditionally quantified by ^1^H-MRS are proton signatures from lipids and proteins. These resonances were once hypothesized to include myelin lipids, which at least one study did not support (239), and suggested to involve methyl and methylene protons from a number of amino acid types within polypeptide chains (240), possibly including those associated with myelin (216). Typically difficult to model and thus quantify and therefore often ignored except in efforts toward removal, these resonances have also exhibited some systematic differences between individuals with and without multiple sclerosis and may thus provide diagnostically useful information.

For example, lipid resonances from 0.8 to 1.5 ppm were qualitatively described as abnormally present in the normal-appearing tissue or lesions of multiple sclerosis patients (93). Resonances suggestive of lipids have additionally been spotted between 0.7 and 1.7 ppm in multiple sclerosis lesions while reported not to have been observed in controls (157). Significant enhancements in broad signals indicative of lipids have also been found in the center of a hyperintense lesion but not in brain tissue lateral to it; furthermore, abnormal lipid signals observed in patients were shown to attenuate to control levels during lesion evolution over the course of several months (24).

In addition, macromolecule resonances concluded to arise from non-lipid sources at 0.9 ppm and 1.3 ppm have exhibited elevations in acute lesions relative to chronic lesions and control white matter, while those at 2.1 and 3.0 ppm appeared to be normal (216), reminiscent of signal increases at 0.9 ppm and 1.3 ppm noted previously in acute plaques (95). While the 0.9 ppm region comprises resonances from both macromolecules and lipids, it has been occasionally found that modeled or measured lipid resonances do not sufficiently account for the amplitude of 0.9 ppm signals observed (95, 216). By contrast, mixed-tissue voxels in sensorimotor but not parietal cortex have exhibited lower macromolecule resonance intensities in multiple sclerosis patients than healthy control (107), though strong peaks from 0.8 to 1.5 ppm have also been observed in some predominantly gray-matter voxels from relapsing-remitting patients but not controls (241). Differences in macromolecular signature between white and gray matter are apparent within individuals with relapsing-remitting disease, whose broad resonances suggestive of macromolecules and lipids at 0.9, 1.2–1.4, and 2.0 ppm have been shown to differ between normal-appearing white matter and gray matter but not between chronic lesions and normal-appearing white matter (224). Other research has found no significant abnormalities in parametrically modeled macromolecule and lipid resonances at 1.7 or 1.2–1.4 ppm among relapsing-remitting normal-appearing white matter, non-enhancing lesions, and control white matter (48).

Because of the relative immobility of protons that inhabit very large molecules like lipids and polypeptides, these species are thought to exhibit shorter transverse relaxation times than those of small-molecule metabolites. This claim is supported by broad frequency-domain signals indicative of fast $T_{2}^{*}$, by their disappearance at high relative to low echo-time acquisitions, and by relaxometry experiments in animals (242) and humans (240, 243). Many ^1^H-MRS studies of multiple sclerosis have been able to benefit from this property to minimize the issue of systematically differing macromolecule and lipid contributions to the spectral baseline by measuring at high (135+ ms) echo times (22, 24–26, 30, 31, 33, 34, 36, 37, 39–45, 47, 50–52, 55–62, 64–66, 71–75, 78–81, 83–86, 89, 94, 96, 98, 121–123, 129, 130, 133, 137, 141, 144, 145, 147, 153, 159, 165, 168, 174, 194, 202, 204, 221–223, 226–231, 244–247). Quantifying metabolites from high-*T*_*E*_ spectra, however, in addition to exhibiting the problems inherent in reduced SNR, exacerbates the potentially confounding effects of *T*_2_ relaxation rates that may systematically differ among experiment groups, as will be discussed in section *T*_2_ Relaxation. Sequences at low echo time may alternatively minimize the lipid or macromolecule signals present in the baseline of acquired spectra by inversion preparation (54, 240, 248). This approach nulls the magnetization associated with *T*_1_ relaxation time constants resembling that expected for macromolecular resonances. Other studies have included measured or modeled lipids and/or macromolecules in their spectral fitting algorithms (28, 48, 95, 107, 136, 144, 167, 249).

Finally, additional research has attempted to account at once for the confounding influences of lipids, macromolecules, and other non-metabolite spectral irregularities by implementing one or more of the following procedures: fitting a baseline modeled by splines (23, 36, 61, 72, 83, 157) as also used in the popular quantification program LCModel (250), or polynomials (48, 64, 74, 94, 95, 130, 133, 225, 251); averaging a subset of FID points (87, 225); alternative semi-parametric or non-parametric baseline modeling schemes (62, 84, 202, 203); or other correction methods (26, 29, 37, 51, 65, 93, 135, 223); in concert with metabolite quantification procedures.

For the few studies that have employed spectroscopy to observe lactate (24, 47, 69, 90, 101, 142, 146, 153, 154, 157–159, 217, 230, 245) or edited GABA (107, 134, 150, 252, 253) in multiple sclerosis, accounting for the potential confounding presence of lipids or other macromolecules is particularly relevant for accurate quantification. The ^1^H-MRS resonance for lactate is a doublet at 1.32 ppm, within the range of both suspected macromolecule and lipid peaks previously observed in multiple sclerosis brain tissue. Though this doublet is quantifiable at *T*_*E*_ high enough for the spectral signatures of overlapping lipids and macromolecules to have already exhibited more significant *T*_2_ decay, the influence of lipid resonances on lactate quantification has been previously reported at *T*_*E*_ as long as 272 ms (174). Similarly, spectrally edited GABA difference signals isolated at 3.01 ppm from creatine, phosphocreatine, and glutathione by frequency-specific editing pulses to the *J*-coupled resonance at 1.89 ppm exhibit, in addition to contamination by the metabolite homocarnosine (254), co-edited macromolecular resonances, motivating the nomenclature “GABA+” in some of the papers that do not employ additional methods to address this confound, as in (150). Unwanted co-editing of macromolecules has been previously minimized by mirroring the editing pulse at the 1.89-ppm GABA peak around the expected frequency location of co-edited macromolecules (253, 255). The effectiveness of this strategy relies on certain assumptions and is vulnerable to radiofrequency pulse displacement and B_0_ field variation, sometimes motivating supplementary nullification of macromolecular resonances by a pre-sequence inversion pulse (252).

### Spectral Quantitation

After ^1^H-MRS data are acquired and preprocessed, the signals associated with each metabolite in a spectrum are quantified as a first step to calculating the concentrations underlying them. This can be a complex task, as single metabolites are often represented by multiple resonances at different frequencies, and most resonances exhibit some degree of overlap with those of other metabolites. In addition, due to peak splitting in some resonances as a result of *J*-coupling, some nuclei present as visually complex shapes that are not only reduced in amplitude, decreasing the probability of effective separation from the noise floor, but also increased in spectral width, increasing the probability of overlap with signals from other molecules. Well-defined singlet resonances, such as those from the methyl protons of the N-acetyl aspartate acetyl moiety at 2.01 ppm, creatine at 3.03 ppm, and choline at 3.2 ppm, may be approximately quantified by integration under properly controlled baseline conditions. Supplementing these estimates with information derived from the additional non-singlet resonances of these metabolites, however, or parsing and quantifying metabolites defined solely by low-SNR multiplets that overlap with resonances from other nuclei, such as myoinositol, can become a mathematically intensive modeling problem. This is especially true when considerations of how to best define the spectral baseline are left flexible by imperfect information.

**Figure 7:**
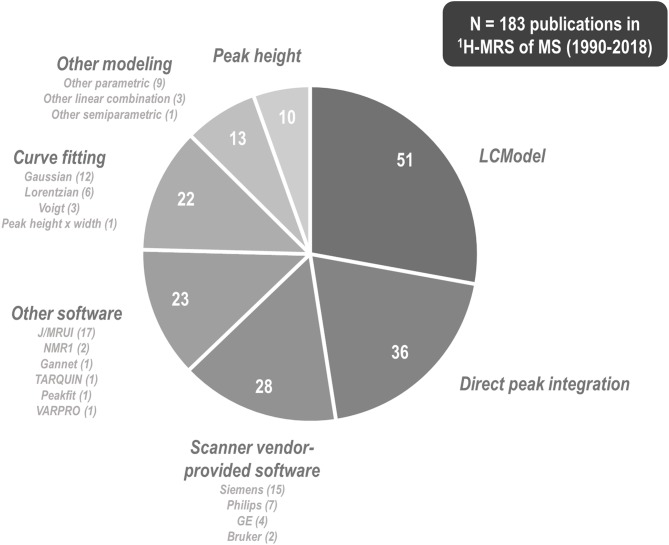
^1^H-MRS research into multiple sclerosis has employed a plurality of methods and software programs for spectral quantification. Among 183 papers published between 1990 and 2018 that describe some method of proton magnetic resonance spectroscopy (^1^H-MRS) quantification for at least one individual with multiple sclerosis (MS), 51 (27.9%) of them used commercial linear combination modeling package LCModel, 36 (19.7%) reported direct integration of key resonances without explicit fitting of basis functions, at least 28 (15.3%) employed software provided by the scanner vendor, 23 (12.6%) reported using other software tools, another 22 (12.0%) fit key peaks by simple analytic functions before integrating, 13 (7.1%) employed alternate semi-parametric and parametric combination modeling and quantification schemes, and 10 (5.4%) quantified spectra by peak height alone. These methodological disparities are significant because different quantification methods may exhibit differential vulnerability to confound by variables like spectral overlap, macromolecule- and lipid-heavy baselines, accuracy of measured or simulated metabolite basis functions, and overall spectral quality.

^1^H-MRS research on multiple sclerosis has historically exhibited a great variety of methods by which to tackle metabolite signal quantification. Among 183 papers published between 1990 and 2018 involving ^1^H-MRS examination of at least one individual with multiple sclerosis, 51 (27.9%) of them used commercial linear combination modeling package LCModel (250); 36 (19.7%) reported direct integration of key resonances without explicit fitting of basis functions; at least 28 (15.3%) employed software provided by the scanner vendor; 23 (12.6%) reported using other software tools (MRUI or JMRUI (256), TARQUIN (257), Gannet (258), and others); another 22 (12.0%) fit key peaks by simplified lineshapes (Gaussian, Lorentzian, or Voigt) before integrating; 13 (7.1%) employed alternate semi-parametric and parametric combination modeling and quantification schemes; and 10 (5.5%) quantified spectra by peak height alone (Figure 7). These methodological disparities are significant because different quantification methods may exhibit differential vulnerability to confound by variables like spectral overlap, macromolecule- and lipid-heavy baselines, accuracy of measured or simulated metabolite basis functions, and overall spectral quality.

For quantification algorithms requiring the fitting of measured, simulated, or analytic metabolite basis functions to acquired spectra, it is important that fit quality be quantified in order to determine the suitability of any outputs for inclusion in further analysis and interpretation. One common metric for fit quality assessment is the Cramér-Rao Lower Bound (CRLB; also called the Cramér-Rao Bound), which defines the lower bound of error standard deviation for a metabolite intensity calculated by a particular model fit, given Gaussian uncertainty surrounding a perfectly informed model (259). CRLB are sometimes reported as percentages of quantified metabolite intensity, which causes them to increase as estimated metabolite levels decrease. As has been argued previously, this is significant because many studies use CRLB as a filter for data quality before statistical analysis, cutting out those data points that do not meet a certain minimum threshold (generally 20–50%) for this metric (260). Similarly, in the event that CRLB exhibit systematic disparities between experimental groups, this practice may be problematic for ^1^H-MRS research into multiple sclerosis by differentially biasing the distribution of data points included in between-group comparisons.

In addition to lower metabolite concentrations, which by necessity will inflate CRLB calculated relative to quantity, decreased SNR, itself also a consequence of reduced metabolite concentration as argued in section Spectral Quality, will also tend to increase the error standard deviations of a model fit and therefore its lower bound. Simulation analysis on diffusion-weighted imaging data has demonstrated that the CRLB on calculated diffusivities decrease with increasing SNR (261). Additionally, examination of experimentally derived metabolite concentrations quantified from nearly identical conditions at 4 T and 7 T showed that average quantification CRLB decreased significantly while SNR nearly doubled from the former to the latter, though increases in spectral dispersion from 4 to 7 T were also cited as a contributing factor to this trend (209). A similar decrease in CRLB with increase in SNR was yielded in a diffusion-weighted ^1^H-MRS study conducted on multiple sclerosis patients at both 3 T and 7 T (262).

Similarly, increased FWHM effectively reduces spectral dispersion and therefore decreases the orthogonality of resonances to which model functions are applied for quantification by fitting. Mathematically, this is expected to increase the CRLB due to increased correlation between the shape of a resonance and that of others in its spectral environment (259). This theory is evidenced in practice by the aforementioned documentations of decreased CRLB with the greater spectral dispersions inherent in higher field strengths, as well as by additional evidence showing that the degree to which calculated CRLB underestimated actual sample variance decreased with spectral line width of the data (236).

As for FWHM and SNR, few studies report data supporting investigation of systematic differences in CRLB among groups differentially affected by multiple sclerosis. In one MRSI study, more than twice as many voxels from MS patients than control failed to meet the maximum CRLB criterion of 20% and were therefore rejected from analysis, though between-group comparisons of CRLB values before rejection were not reported (57). Another study reported much higher glutamate-glutamine CRLB in lesions and normal-appearing white matter than in cortical gray matter, attributed to lower measured concentrations in the first two tissue types, though, again, no cross-sectional analysis against control values was presented (224).

## Metabolite Quantification

### Correction for Cerebrospinal Fluid Volume and Voxel Water Molarity Estimation

Multiple sclerosis has been associated with multiple patterns of cortical tissue atrophy relative to age- and sex-matched controls (263). The resultant influence of differential voxel CSF on the absolute concentrations of brain metabolites can be characterized by a few methods. These include image segmentation (62, 84, 99–104, 107, 115, 117–119, 123, 124, 131, 134, 146, 149, 150, 162, 164, 167, 173, 188, 191, 203, 223, 233, 252, 253), compartmentalization by multiexponential modeling of *T*_2_ (67, 69, 70, 88, 92, 105, 106, 110, 116, 120, 132), or *T*_1_ relaxometry (135).

Less well documented are the potential effects of multiple sclerosis on brain tissue water molarity, important for using water as an internal metabolite quantification reference. Previous examination of this question has demonstrated no significant difference between individuals with and without relapsing-remitting multiple sclerosis in brain tissue water content in the thalamus (70) or normal-appearing white matter (120) but has shown evidence of greater tissue water content in contrast-enhancing lesions than in healthy control brain (116) and reductions in cortical gray-matter water fraction of both relapsing-remitting and secondary progressive patients relative to control (88). While some previous spectroscopy research on multiple sclerosis has used referencing by internal water for metabolite quantification (99, 103, 105, 107, 117–119, 124, 134, 146, 149, 150, 162, 174, 189, 224, 253, 254), the possible confounds of disease-based differences in tissue water molarity can be minimized through use of external referencing for metabolite quantification through water (70, 88, 120), N-acetyl aspartate (82, 109, 111–114, 122, 123, 126, 137, 186, 187, 192, 193, 203, 207, 208, 264, 265), acetate (167), or a mixture of reference metabolites (22, 29, 62, 84, 92, 100, 104, 191, 202, 233). While external referencing via scanning a phantom of known composition carries with it the need to additionally account for differences with the human head in radiofrequency coil load, this factor has been addressed experimentally by scanning the external reference together with the human participant and accounting for signal differences in metabolite and reference voxel location (88, 120); by treating a voxel of CSF, with associated corrections for B_1_ field differences, as an internalized external reference of pure water (50, 135); or by regressing against the CSF contents of multiple voxels to calculate a pure water reference for the brain regions under study (162, 188). Differences between human and phantom in the rate of signal decay by *T*_1_ and *T*_2_ relaxation must be additionally corrected for sequences of non-zero echo time and finite repetition time by extrapolation to 0 echo time and infinite repetition time based on either empirical assessment and/or use of literature values of *T*_1_ and *T*_2_.

### Partial Volume Correction for Lesions

Multiple sclerosis lesions have been shown to exhibit multiple metabolic differences from non-lesioned or healthy brain tissue. Lesions of varying enhancement and chronicity have exhibited decreases in N-acetyl aspartate referenced to creatine (25, 34, 60, 78, 144) or otherwise (78, 117, 174); creatine-referenced choline (93, 143), and creatine (117) as well as increases in creatine-referenced choline (25, 34) relative to multiple sclerosis normal-appearing white matter. In particular, acute contrast-enhancing white-matter lesions have demonstrated apparent decreases in N-acetyl aspartate (117) as well as increases in creatine-referenced inositol (24). Chronic or *T*_1_-hypointense lesions have exhibited reduced N-acetyl aspartate (22, 115, 137), decreased creatine (22, 137), decreased (137) or increased (115) choline, and increased creatine-referenced myoinositol (148) or no metabolic differences (224) relative to multiple sclerosis normal-appearing white matter. *T*_1_-isointense lesions have demonstrated increases in creatine and choline (115) or no difference (137) in creatine, choline, or N-acetyl aspartate relative to multiple sclerosis normal-appearing white matter.

A number of studies control for these potentially confounding differences by avoiding visible lesions in the placement of a spectroscopy voxel within “normal-appearing” brain matter and/or applying manual or automatic image segmentation to the voxel once analyzed in order to numerically correct for lesion partial volume in statistical models. These two methods are limited, however, by the resolution and signal contrast of the imaging sequences employed to identify lesions ideally in the same session as spectroscopic data acquisition. ^1^H-MRS studies employing post-acquisition lesion identification have used a variety of imaging sequences for this purpose, including *T*_1_-weighted imaging with (27, 87, 118, 119, 131, 163, 216, 247) and only without (61, 65, 66, 83, 100, 102, 134, 149, 188, 252, 253) injectable contrast, FLuid-Attenuated Inversion Recovery (FLAIR) (103, 107, 121, 167, 168, 191, 233), proton density imaging (52, 61, 65, 83, 118, 121, 131, 163, 249), *T*_2_ weighting (27, 52, 61, 65, 66, 83, 103, 107, 108, 117–119, 121, 124, 134, 149, 163, 247), double inversion recovery (134), and alternative sequences applying both fluid attenuation for CSF nulling and magnetization transfer contrast for signal reduction in normal tissue relative to lesions (87, 146). Each of these methods uncovers a limited range of lesion types. While *T*_1_-weighted imaging without contrast may aid in the identification of chronic, hypointense *T*_1_-weighted “black holes,” for example, without the injection of contrast agent it is of limited use to identify active lesions marked by increased blood-brain barrier permeability and acute inflammation. On the other hand, *T*_2_-weighted FLAIR may be used to identify a more general range of lesions, but this is a broad category of heterogeneous cases whose metabolic signatures may differ based on *T*_1_-weighted contrast intensity. With this fact under consideration, a large proportion of ^1^H-MRS studies on multiple sclerosis have used multiple imaging methods in lesion identification and segmentation (27, 52, 61, 65, 66, 83, 87, 118, 119, 121, 131, 134, 149, 163, 247).

The most rigorous controls for lesion partial volume correction would take into account the range of heterogeneous lesion types potentially present in the brain tissue of an individual with multiple sclerosis. The metabolic composition of *T*_1_-weighted lesions has been shown to depend on, for example, their signal intensity relative to the surrounding tissue (22, 115, 233, 265); disparate patterns of metabolic abnormality have also been observed in different *T*_1_-weighted lesions depending on whether they are considered to be acute or chronic (77, 92, 148, 216) or contrast-enhancing vs. non-enhancing (245). It has furthermore been argued that active demyelinating lesions may also be characterized based on the presence and spatial arrangement of certain cell types, signaling molecules, and other features of immunopathology (266–269), classifications that have yet to be associated with MR-visible features. In addition, MR-invisible lesion-related biochemical heterogeneity may also be present within normal-appearing white matter: For example, despite not being distinguishable by imaging, normal-appearing white matter that later developed new lesions at 6-month follow-up has reportedly demonstrated higher creatine and choline than corresponding tissue that did not (233). Similarly, post-mortem verification of imaging data has demonstrated that upwards of 80% of gray-matter lesions may be invisible to even double-inversion-recovery imaging sequences (270, 271).

### Gray Matter-White Matter Composition

Gray matter derives its name from the gray color taken by the oxidation of vascularized tissue after formalin fixation (272) and largely constitutes the cell bodies of neurons as well as surrounding glial cells. White matter, called so because of the white lipids comprising a major component thereof, represents the myelinated or oligodendrocyte-sheathed axons projecting from them. While both tissue types comprise neurons, oligodendrocytes and precursors, astrocytes, microglia, endothelium, vascular tissue, extracellular matrix components, and, in the case of multiple sclerosis lesion activity, immune cells like T cells as well as activated microglia and macrophages (273), these two tissue classifications exhibit distinctive metabolic signatures. Previous research has demonstrated, for example, significantly higher concentrations of N-acetyl aspartate, creatine, and choline in gray than in white matter (274). Differences in the relative proportions of gray and white matter in mixed-tissue voxels between individuals with and without multiple sclerosis may consequently obscure disease-associated disparities in metabolite concentration within each tissue type. The apparent diffusion coefficients (275) as well as *T*_1_ and *T*_2_ relaxation times of some major metabolites (276) have also been shown to differ between gray and white matter, implying that any such signal weighting imposed by a pulse sequence on these metabolites as quantified from a particular voxel will be influenced by its gray-white matter composition.

In addition, while ample evidence of abnormal concentrations of N-acetyl aspartate (54, 70, 83, 94, 100, 103, 104, 106, 115, 118, 119, 121, 122, 131–133), glutamate-glutamine (119, 131), choline (119, 122, 144), creatine (133), and inositols (106) in the gray matter of individuals with multiple sclerosis refutes the outdated notion that multiple sclerosis is a white-matter disease, some evidence from the ^1^H-MRS literature alone suggests differences in its relative effects on gray and white matter. While gray-white matter composition has been found to be similar in posterior cingulate cortex, medial prefrontal cortex, and left hippocampus between healthy controls and individuals with relapsing-remitting multiple sclerosis (150), as well as in a slab of voxels from central brain between controls and primary progressive multiple sclerosis (131), other research has shown decreases in total brain white but not gray matter fraction in secondary progressive multiple sclerosis relative to control (134). Research in imaging has suggested that rates of neurodegeneration in multiple sclerosis may differ not only by region (277) but also by tissue type (278–280), predicting voxel composition differences from control that could affect the spectroscopic analysis thereof.

Researchers using ^1^H-MRS to study multiple sclerosis have attempted to contend with this potential confound in a few different ways. Foremost among these is to restrict the voxel(s) from which spectra are acquired to either white or gray matter [see (23, 40, 90, 92, 105) for just a few examples], though this method reduces the maximum potential SNR via constraints to voxel size as well as promotes partial volume contamination in the event that measured volumes are not segmented and selectively excluded post-acquisition. Other investigations have used MRSI to measure mixed-tissue volumes and then segmentation to classify them as either white or gray matter (131, 233). A variant on this approach has been to regress metabolite signals from multiple voxels against percent of white or gray matter to derive “pure” white or gray-matter estimates (102, 104, 163, 164). Still others have acquired data from mixed-tissue voxels and included voxel composition as covariates in statistical analysis [for example, (103, 118)].

### T_1_ Relaxation

Longitudinal or spin-lattice relaxation time constant *T*_1_ describes the nucleus-specific rate at which proton spins excited by a radiofrequency pulse relax exponentially back to thermodynamic equilibrium, affecting the amount of magnetization available for re-excitation and subsequent measurement (281). As *T*_1_ depends in part on features of the local chemical environment, consistent disparities in tissue *T*_1_ between individuals with and without multiple sclerosis may exist and systematically influence investigations of apparent metabolic abnormality in the former.

Brain tissue affected by multiple sclerosis may exhibit abnormal *T*_1_ relaxivity that NMR analysis has suggested could vary with tissue water content (282). Whole-brain mapping of water *T*_1_ has found, for example, higher modal water *T*_1_ in normal-appearing white matter as well as gray matter in MS patients relative to control, with secondary progressive patients exhibiting histogram peaks at higher *T*_1_ values than those of with either relapsing-remitting or primary progressive disease (283). Other research has associated longer *T*_1_ relaxation in multiple sclerosis lesions with lower concentrations of N-acetyl aspartate and increased inositol, potentially reflective of axonal damage and gliosis (265). This result was recapitulated in lesions, in which multiple sclerosis patients demonstrated prolongations of water *T*_1_ relative to control that correlated inversely with measured concentrations of N-acetyl aspartate, choline, and creatine (22), while another study found that *T*_1_ correlated positively with N-acetyl aspartate in patients with few focal lesions but negatively with choline in those with many (284). Water *T*_1_ values have been shown to increase in gray matter for progressive but not relapsing-remitting multiple sclerosis patients relative to healthy control, as well as in enhancing relative to non-enhancing lesions within both relapsing-remitting and progressive multiple sclerosis patients (285). Quantitative MRI (qMRI) measurements of water *T*_1_ have additionally demonstrated higher values than control in diffusely abnormal white matter even in the absence of *T*_1_ abnormality in normal-appearing white matter, further supporting the idea that the degree of relaxivity difference from control can be tissue-specific (284). Limited evidence suggests that multiple sclerosis pathology may differentially affect the *T*_1_ of water, dominated by a fluid compartment, and metabolites, typically bound to tissue compartments: One study of eight patients and eight controls, for instance, indicated that occipital and parietal lesions exhibited increases in water *T*_1_ but decreases in choline *T*_1_ relative to control white matter, even while the *T*_1_ of creatine, N-acetyl aspartate, and myoinositol did not change significantly (249).

The magnitude of influence by *T*_1_ relaxation on an experiment can be adjusted by altering long sequence intervals like repetition time *T*_*R*_, inversion time *T*_*I*_, and mixing time *T*_*M*_. A sizeable portion of ^1^H-MRS research on multiple sclerosis attempts to skirt the issue of *T*_1_ effects on metabolite quantification by employing pulse sequences with long repetition times. This strategy, however, is imperfect, as the *T*_1_ of not only water (286) but also metabolites (276) can be as long as one second or more in the brain, requiring repetition times of several seconds to avoid significant *T*_1_ weighting given the rule of thumb that as much as five times a species' *T*_1_ is required for what is considered an adequate return to equilibrium. Some studies reporting absolute concentration values attempt to explicitly consider the effects of *T*_1_ by including published *T*_1_ values for metabolites and/or water (29, 80, 91, 94, 96, 98, 100, 102, 103, 105, 107, 122, 123, 129, 130, 133, 135, 137, 149, 162, 167, 188, 191, 202, 229, 233, 252, 253) in their quantitation algorithms. This approach, however, does not necessarily account for potential disparities in relaxivity based on region, sequence, disease state, and other experiment-specific variables. Some research has attempted to maximize the applicability of literature values for *T*_1_ by taking into account voxel composition of gray matter, white matter, and/or CSF and water and/or metabolite *T*_1_ differences thereof (100, 107, 149, 162, 252, 253). Few studies to date empirically correct for these potential confounds by estimating them in-house for the cohort under study (92, 163, 164, 249, 265), examining their potential effects on the measurements acquired (55), or applying disease-specific *T*_1_ values from the literature to the experimental groups at hand (107).

### T_2_ Relaxation

Transverse or spin-spin relaxation time constant *T*_2_ is an exponential decay constant that describes the rate at which the transverse magnetization of a proton sample shrinks over time as a result of dephasing from interactions among spins (pure *T*_2_) and spatial B_0_ field variation across the measurement volume (described by *T*_2_', the synthesis of which with *T*_2_ is called $T_{2}^{*}$). Like *T*_1_, *T*_2_ is a nucleus- and tissue-specific property that affects the apparent concentrations of measured metabolites by influencing the signal available for acquisition and quantification (281).

Previous research suggests that water *T*_2_ differs between individuals with and without multiple sclerosis. For example, multiple sclerosis normal-appearing white matter, diffusely abnormal or “dirty” white matter, non-enhancing lesions, and enhancing lesions have all demonstrated higher water *T*_2_ than the normal white matter of controls. Among lesions, the magnitude of this increase appeared to depend on contrast type, with black hole lesions exhibiting the highest water *T*_2_ and non-enhancing isointense or enhancing lesions exhibiting the lowest (287). In addition, relative to diffuse lesions, focal multiple sclerosis spinal lesions have demonstrated increased water *T*_2_, which correlated negatively with choline measured in the voxel (29), a finding corroborated in another study that found increases in both the coefficient and value of the long component of biexponential water *T*_2_ in contrast-enhancing multiple sclerosis ring lesions relative to control, the value of which correlated inversely with measured N-acetyl aspartate and creatine concentrations (116). Other research found that normal-appearing white matter water *T*_2_ correlated positively with N-acetyl aspartate concentration in relapsing-remitting multiple sclerosis patients with few visible lesions but not in those with many (284).

Observations of metabolite *T*_2_ alterations in multiple sclerosis, however, have been limited. Research in mostly gray matter mixed-tissue voxels has found minimal effect of relapsing-remitting and secondary progressive multiple sclerosis on metabolite *T*_2_ despite significant reductions in water *T*_2_ (88). Similarly, metabolite *T*_2_ were comparable between multiple sclerosis lesions and normal-appearing white matter despite expected increases in water *T*_2_ evinced by changes in image contrast; reductions from control in multiple sclerosis gray and white matter *T*_2_ estimates for N-acetyl aspartate, creatine, and choline also fell within the range of intra-subject variation (232). One study comparing apparent metabolite ratios measured by ^1^H-MRS at short and long echo times has suggested potential decreases in choline relative to creatine *T*_2_ in white-matter voxels that contained 25–50% lesion (55).

Postmortem relaxometry of spinal cord lesions supports the notion that pathological increases in lesion water *T*_2_ may reflect the degree of local demyelination (288), which may increase the fraction of voxel water protons available to diffuse through the extracellular space and contribute to a longer *T*_2_ component (116). On the other hand, observed decreases in metabolite *T*_2_ have been tentatively attributed to potential reductions in brain cell size from osmotic flight of intracellular water and compression by a denser local population of glia (191, 232), increasing the frequency of dephasing interactions among intracellular metabolite spins. Resonance-invariant change in *T*_2_ with disease is therefore not a physical necessity.

As previously discussed, macromolecules and lipids are considered to exhibit faster *T*_2_ decay than small-molecule metabolites. The presence of these compounds may therefore disproportionately influence metabolite concentrations at lower echo times, therefore confounding the *T*_2_ relaxation constants estimated from them. If macromolecular baselines are expected to differ between two groups, then, estimated *T*_2_ may also differ. This possibility has been raised as a potential confound underlying the investigation of age-related effects on metabolite *T*_2_ (289) and may thus also contribute to any apparent abnormalities in relaxivity found in multiple sclerosis if not properly controlled. The influence of this factor may be minimized, as mentioned in section Macromolecule and Lipid Contributions to the Spectroscopic Baseline, by building pulse sequences that properly null these signals for short-echo-time measurements or by accounting for them in subsequent analysis.

Since *T*_2_ represents the decay of proton signal due to dephasing following spin excitation, its influence on measured signals increases with echo time. One sequence for whole-brain N-acetyl aspartate measurement therefore minimizes this issue by effectively acquiring at zero echo time (113, 114, 125–128, 186, 187, 192, 193, 207, 208). Another way of minimizing the confounding influence of *T*_2_ on apparent metabolite concentrations has been, as in controlling for *T*_1_, to account for the *T*_2_ of water and/or metabolites by applying values from the literature (80, 84, 94, 96, 98, 105, 122, 123, 129, 130, 133, 137, 167, 188, 191, 202, 203, 227, 229), including to calculations accounting for the particular tissue composition of each voxel (107, 149, 162, 233, 252, 253). More precision may be offered by estimating *T*_2_ directly in the system at hand (29, 67, 70, 88, 92, 100, 101, 107, 116, 120, 135), though for maximum utility any such approach would be applied to all compounds under study.

### B_1_ Transmit and Receive Sensitivity

In ^1^H-MRS, B_1_ refers to the strength of local magnetic fields imposed by radiofrequency waves emitted by either the transmit coil (transmit or ${\text{B}}_{1}^{+}$) or protons precessing on the transverse plane (receive or ${\text{B}}_{1}^{-}$). In addition to contributing to variance among participants or between metabolite signals acquired from *in vivo* and phantom scans, non-uniform B_1_ has been shown to disproportionately affect the intensities of coupled relative to singlet resonances (290). This confound is difficult to unravel in data post-processing and may impact the quantification of molecules like N-acetyl aspartate, glutamate, glutathione, and GABA, especially when they are referenced to metabolites with non-coupled spin systems, like creatine.

Both transmit and receive B_1_ inhomogeneity may also play a role in studies in which metabolites are quantified relative to a signal external to the voxel used for spectroscopy, such as the water signal from the ventricles or a separately measured metabolite phantom. Because individuals with multiple sclerosis can exhibit wider ventricles than those without (291), one may imagine a scenario in which periventricular cortical voxels are systematically located more peripherally in those with more progressed disease. Depending on the field profiles of the transmit pulses and receive coils used, such an offset may lead to either over- or underestimation of the metabolites at hand in this cohort, especially at higher field strengths at which increased Larmor frequency necessitates the use of radiofrequency pulses of a wavelength shorter than the diameter of the human skull. For example, a significant increase in SNR at the brain center relative to the periphery has been previously reported using a volume coil at 7 Tesla; correspondingly, peripheral B_1_ was measured to be more than 40% lower than that in the center (292). Correspondingly, post-acquisition correction for the effects of B_1_ transmit inhomogeneity on ^1^H-MRSI metabolite acquisitions at 7 T was shown to decrease the apparent concentration of NAA in the center of a phantom and increase it on the periphery (104). Such disparities must be accounted for in any quantification scheme that does not employ an internal standard within the same voxel as the metabolite in question, such as in external referencing to a phantom.

The possibility that multiple sclerosis pathology itself affects B_1_ field strength has received limited treatment in the proton spectroscopy literature, though it has been empirically considered in a cross-sectional comparison of chemical exchange saturation transfer (CEST) measurements of glutamate in the spinal cord of multiple sclerosis patients vs. controls, in which the two groups were shown to exhibit no differences in flip angle due to disparities in ${\text{B}}_{1}^{+}$ penetrance (293).

Heterogeneity in B_1_ receive or ${\text{B}}_{1}^{-}$ profiles has sometimes been corrected in post-acquisition spectral preprocessing using the sensitivity profile of the coil (64, 84). ${\text{B}}_{1}^{+}$ maps have similarly been measured for later correction of spectral intensities due to local differences in induced flip angle (104, 162, 188). In addition, differences in transmit and receive B_1_ sensitivity between signal acquisitions *in vivo* and in the phantoms used for absolute metabolite quantification have been estimated to enable appropriate comparison of *in vivo* relative to phantom signal ratios (70, 116). Finally, previous examinations of multiple sclerosis using ^1^H-MRS exhibit limited use of adiabatic pulses to improve B_1_ homogeneity (188).

## Outlook and Conclusions

Since its first application in 1946, ^1^H-MRS has offered a safe and flexible means of noninvasively estimating the concentrations of various small-molecule metabolites in living tissue, including the brain. Though over 190 original research and case reports using ^1^H-MRS to examine the central nervous system metabolic signatures of multiple sclerosis have been published since 1990, the field continues to lack knowledge of a single metabolite alteration that can enable the identification of multiple sclerosis with sufficient sensitivity and specificity for appropriate clinical use.

An abundance of evidence exists, for example, that cortical N-acetyl aspartate in both gray and white matter drops with relapsing-remitting (Figure 1), secondary progressive (Figure 2), and primary progressive (Figure 3) multiple sclerosis, but even this effect, the most robust across the ^1^H-MRS literature on multiple sclerosis, is subtle and not always reproduced. Among 64 publications reporting comparisons with control in this metric for individuals with unspecified or mixed, relapsing-remitting, or progressive phenotypes in voxels that were not predominantly lesions, 22 publications did not reject the null hypothesis for comparisons in at least one tissue type, and 11 reported only null results, for creatine-referenced N-acetyl aspartate (Table 1).

**Table 1:** Studies using ^1^H-MRS to examine difference from control in tNAA/tCr ratios in brain or spine non-lesion tissue.

**References**	**MS**	**Tissue**	**Effect**	**References**	**MS**	**Tissue**	**Effect**
Aboul-Enein et al. (80)	SP	NAWM	↓ in MS	Pan et al. (54)	R	mixed	↓ in MS
Aboul-Enein et al. (80)	R	NAWM	NS	Parry et al. (74)	R	mixed	↓ in MS
Anik et al. (41)	M	WM	↓ in MS	Pascual et al. (141)	R	NAWM	NS
Anik et al. (41)	M	NAWM	↓ in MS	Pelletier et al. (85)	PP	mixed, supratentorial	↓ in MS
Arnold et al. (47)	M	mixed	↓ in MS	Pelletier et al. (85)	PP	mixed, excluding central	↓ in MS
Bagory et al. (84)	SP	mixed	↓ in MS	Pelletier et al. (85)	PP	mixed, central	↓ in MS
Bagory et al. (84)	PP	mixed	↓ in MS	Pelletier et al. (85)	SP	mixed, supratentorial	↓ in MS
Bagory et al. (84)	R	mixed	NS	Pelletier et al. (85)	SP	mixed, excluding central	↓ in MS
Bellmann-Strobl et al. (65)	R	NAWM	↓ in MS	Pelletier et al. (85)	SP	mixed, central	↓ in MS
Brass et al. (43)	M	NAWM	↓ in MS	Pelletier et al. (85)	R	mixed, supratentorial	NS
Caramanos et al. (83)	SP	GM	↓ in MS	Pelletier et al. (85)	R	mixed, excluding central	NS
Caramanos et al. (83)	R	GM	NS	Pelletier et al. (85)	R	mixed, central	NS
Casanova et al. (204)	R	NAWM, peduncles	NS	Pelletier et al. (89)	PP	mixed	↓ in MS
Casanova et al. (204)	R	NAWM, pons	NS	Pokryszko-Dragan et al. (63)	R	mixed	↓ in MS
Cucurella et al. (78)	SP	NAWM	NS	Pokryszko-Dragan et al. (63)	R	WM	↓ in MS
Cucurella et al. (78)	PP	NAWM	↓ in MS	Reddy et al. (59)	R	WM	↓ in MS^*^
Davie et al. (24)	M	NAWM	↓ in MS	Rooney et al. (37)	M	NAWM	↓ in MS
De Stefano et al. (86)	RP	mixed	↓ in MS^*^	Ruiz-Peña et al. (143)	R	NAWM	NS
De Stefano et al. (36)	M	WM	↓ in MS	Sarchielli et al. (53)	R	NAWM	NS
De Stefano et al. (36)	R	WM	↓ in MS	Sarchielli et al. (88)	SP	mixed	↓ in MS
De Stefano et al. (36)	SP	WM	↓ in MS	Siger-Zajdel et al. (44)	M_sp_	NAWM	↓ in MS
De Stefano et al. (72)	R	mixed	↓ in MS	Siger-Zajdel et al. (44)	M_f_	NAWM	↓ in MS
De Stefano et al. (61)	R	WM	↓ in MS	Staffen et al. (58)	R	mixed	↓ in MS
D'Haeseleer et al. (42)	M	NAWM	↓ in MS	Staffen et al. (58)	R_nl_	NAWM	NS
Duan et al. (64)	R	WM	↓ in MS	Staffen et al. (58)	R_l_	WM	↓ in MS
Fu et al. (52)	SP	NAWM	↓ in MS	Steen et al. (81)	P	NAWM	↓ in MS
Fu et al. (52)	R	NAWM	↓ in MS	Steen et al. (39)	M	NAWM	↓ in MS
Fu et al. (60)	SP	WM	↓ in MS	Suhy et al. (50)	PP	NAWM	↓ in MS
Fu et al. (60)	R	WM	↓ in MS	Suhy et al. (50)	R	NAWM	↓ in MS
Hannoun et al. (62)	SP	WM	↓ in MS	Sun et al. (68)	R	NAWM, frontal	↓ in MS
Hannoun et al. (62)	PP	WM	↓ in MS	Sun et al. (68)	R	NAWM, parietal	↓ in MS
Hannoun et al. (62)	R	WM	↓ in MS	Sun et al. (68)	R	NAWM, parietal-occipital	↓ in MS
Husted et al. (30)	M	NAWM	↓ in MS	Takeuchi et al. (51)	R	NAWM	↓ in MS
Kimura et al. (32)	M	NAWM	NS	Tartaglia et al. (25)	M	NAWM	↓ in MS
Leary et al. (82)	PP	NAWM	↓ in MS	Tedeschi et al. (34)	M	NAWM	↓ in MS
Maffei et al. (76)	R	spine	↓ in MS^*^	Téllez et al. (75)	R_hf_	mixed, lentiform nucleus	↓ in MS
Maffei et al. (76)	SP	spine	NS^*^	Téllez et al. (75)	R_hf_	WM, frontal	NS
Mathiesen et al. (144)	R	GM	NS	Téllez et al. (75)	R_lf_	mixed, lentiform nucleus	NS
Mathiesen et al. (144)	R	mixed	NS	Téllez et al. (75)	R_lf_	WM, frontal	NS
Mathiesen et al. (144)	R	NAWM	NS	Tourbah et al. (40)	M	NAWM	↓ in MS
Matthews et al. (55)	M	NAWM	NS	Tourbah et al. (46)	M	NAWM	↓ in MS
Matthews et al. (71)	SP	mixed	↓ in MS	Tourbah et al. (46)	R	NAWM	NS
Matthews et al. (71)	R	mixed	↓ in MS	Tourbah et al. (46)	SP	NAWM	↓ in MS
Narayanan et al. (73)	SP	mixed	↓ in MS	Tourbah et al. (79)	R	NAWM	NS
Narayanan et al. (73)	R	mixed	↓ in MS	Tourbah et al. (79)	SP	NAWM	↓ in MS
Narayana et al. (87)	PP	mixed	↓ in MS	van Walderveen et al. (22)	M	NAWM	↓ in MS
Obert et al. (172)	SP	NAWM	NS	Vingara et al. (48)	R	NAWM	↓ in MS
Obert et al. (172)	R	NAWM	NS	Vrenken et al. (69)	PP	NAWM	↓ in MS
Oguz et al. (148)	R	NAWM	NS	Vrenken et al. (69)	SP	NAWM	↓ in MS
Oh et al. (45)	M	NAWM	↓ in MS	Vrenken et al. (69)	R	NAWM	↓ in MS
Oh et al. (45)	PP	NAWM	↓ in MS	Wattjes et al. (67)	R	NAWM	↓ in MS
Oh et al. (66)	SP	NAWM, c.c.	↓ in MS	Wood et al. (38)	M	NAWM	↓ in MS
Oh et al. (66)	SP	NAWM, central	↓ in MS	Wu et al. (145)	R	mixed	NS
Oh et al. (66)	SP	NAWM, not c.c.	NS	Wylezinska et al. (70)	R	GM	↓ in MS
Oh et al. (66)	R	NAWM, c.c.	↓ in MS	Wylezinska et al. (70)	R	NAWM	↓ in MS
Oh et al. (66)	R	NAWM, central	NS	Yetkin et al. (56)	R	NAWM	NS
Oh et al. (66)	R	NAWM, not c.c.	NS	Zaini et al. (57)	R_hf_	WM	↓ in MS
Pan et al. (54)	R	GM	↓ in MS	Zaini et al. (57)	R_lf_	WM	↓ in MS
Pan et al. (54)	R	WM	↓ in MS				

* Single-subject MS case report.

*MS: multiple sclerosis; P: progressive; SP: secondary progressive; PP: primary progressive; R: relapsing-remitting; M: mixed or unspecified MS phenotype(s); c.c.: corpus callosum; M_sp_: sporadic MS; M_f_: familial MS; R_nl_: relapsing-remitting with no lesions in region of interest; R_l_: relapsing-remitting with lesions in region of interest; R_hf_: relapsing-remitting with any or high fatigue; R_lf_: relapsing-remitting with no or low fatigue*.

**Figure 8:**
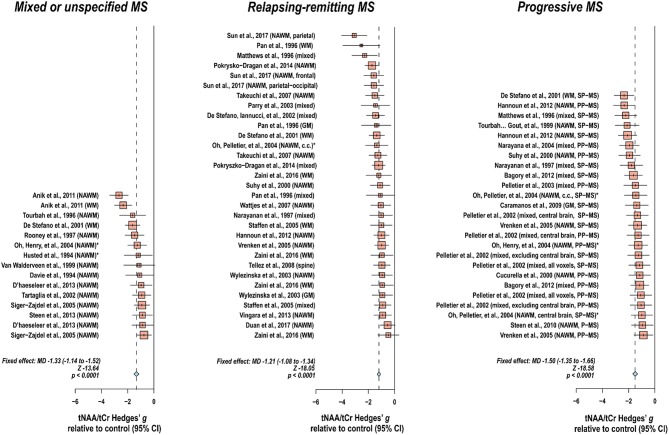
Effect sizes for disease state by multiple sclerosis phenotype in creatine-referenced N-acetyl aspartate as measured by ^1^H-MRS. Among 64 publications reporting comparisons with control in proton magnetic resonance spectroscopy (^1^H-MRS) N-acetyl aspartate referenced to creatine for individuals with unspecified or mixed, relapsing-remitting, or progressive MS phenotypes in non-lesion voxels, 22 publications did not reject the null hypothesis for comparisons in at least one tissue type (Table 1). Among those reporting significant between-group effects of disease on this parameter, the largest effect size from meta-analysis of studies conducted on individuals with various MS phenotypes was for comparisons involving progressive MS cohorts (including unspecified progressive, secondary progressive, and primary progressive), with a standardized mean difference (Hedges' *g*) of −1.50 in 25 comparisons over 16 publications reporting group sizes, means, and labeled standard deviations or errors for this metabolite in voxels that were not predominantly lesions. *Standard deviations calculated from group sizes and standard errors. MD: standardized mean difference, reported as Hedges' *g*; CI: Confidence interval; NAWM: normal-appearing white matter; WM: white matter; GM: gray matter; SP-MS: secondary progressive multiple sclerosis; PP-MS: primary progressive multiple sclerosis; c.c.: corpus callosum.

Among those studies reporting significant between-group effects of disease on creatine-referenced N-acetyl aspartate, the largest effect size from a fixed-effects model (189) of studies conducted on individuals with various multiple sclerosis phenotypes was for comparisons involving progressive multiple sclerosis cohorts (including mixed or unspecified progressive, secondary progressive, and primary progressive), with a standardized mean difference (Hedges' *g*) of −1.50 in 25 comparisons over 16 publications reporting group sizes, means, and labeled standard deviations or errors for creatine-referenced N-acetyl aspartate as measured by ^1^H-MRS in voxels that were not predominantly lesions (Figure 8). This effect size is comparable to the standardized mean differences between secondary progressive cohorts and control, also reported as Hedges' *g*, found by Caramanos et al. (294) for absolute total N-acetyl aspartate concentrations reported in comparisons over studies on normal-appearing white matter (*g* = −0.96, N = 7, *p* = 0.039) and normal-appearing gray matter (*g* = −1.29, N = 4, *p* = 0.0522); our estimate is likely slightly larger than these published values due to its inclusion of studies examining primary progressive patients as well as white-matter voxels containing some lesioned tissue.

In contrast to this broad range of findings, ^1^H-MRS detection of 2-hydroxyglutarate for brain tumor characterization, a rare category of ^1^H-MRS clinical applications for which insurance reimbursement is sometimes considered (14), has previously demonstrated in at least one analysis all-or-nothing association with the presence of isocitrate dehydrogenase mutation status in gliomas (295), supporting, according to a 2018 meta-analysis of 14 studies, a pooled sensitivity of 95% (296). Even this application of ^1^H-MRS, however, is suboptimal for patients whose tumors have already undergone resection or radiotherapy and still not considered definitive in the absence of a biopsy (297).

In addition to appearing to lack a sufficiently reproducible effect size for application to rule-based diagnostics of single individuals, reductions in N-acetyl aspartate in normal-appearing or lesioned brain matter are also not specific to multiple sclerosis, also having been found in diabetes mellitus cortex (298), lupus gray and white matter (299), and HIV basal ganglia (300). Moreover, among conditions demonstrating lesion activity on magnetic resonance imaging for which ^1^H-MRS might serve as an auxiliary toward differential diagnosis, N-acetyl aspartate has similarly been reported to decrease in, among others, acute disseminated encephalomyelitis lesions (301) and lesioned tissue in patients with cerebral small vessel disease (SVD) (302). Along the same lines, one classification analysis of demyelinating lesions and gliomas based on N-acetyl aspartate alone incorrectly classified every demyelinating lesion, even while it demonstrated higher than chance accuracy to differentiate among glioma types (230). One notable exception to the low specificity of N-acetyl aspartate reductions to multiple sclerosis may be in the differentiation between multiple sclerosis and neuromyelitis optica (NMO), as NMO has demonstrated increased N-acetyl aspartate concentration relative to multiple sclerosis while not differing from control in white matter (64), recapitulating a previous report of normalcy in both white and gray matter (303). Additionally, as mentioned, a limited number of studies comparing multiple sclerosis subtypes have reported evidence suggesting greater decreases in N-acetyl aspartate in secondary progressive than relapsing-remitting white matter (36, 79, 80, 105, 120). Despite these counterexamples, taken together, the state of the current literature suggests that even the most reproduced finding of metabolic abnormality in the ^1^H-MRS literature on multiple sclerosis is currently not widely applicable as a diagnostic biomarker.

In a recent survey of the *in vivo* proton magnetic resonance spectroscopy community, “inconsistent or unreliable data quality/reproducibility” was weighted most heavily as the largest practical barrier to the wider clinical application of MRS. Evidence-based standards for generating high-quality spectroscopic data, as those reported by the same work (234), thus appear to represent a welcome point of methodological improvement that may bear fruit for improving the sensitivity and specificity of potential biomarkers derived from ^1^H-MRS, not just for multiple sclerosis diagnosis but for a variety of clinical applications. These include, among others, maximally short *T*_*E*_ and long *T*_*R*_ to minimize signal degradation and confound by relaxation effects, use of adiabatic pulse sequences like LASER, and spectral quantification based on model fits rather than reliance on scanner software or direct integration of spectral lineshapes (234).

Also promising to multiple sclerosis diagnostics may be the determination of a potential disease-specific signature of subtle alterations in many metabolites. While one aforementioned study boasted a 100% failure rate at acute demyelinating lesion identification when it employed a predictive algorithm built using linear discriminant analysis of N-acetyl aspartate concentration alone, the inclusion of additional inputs from choline, creatine, lactate, and lipids enabled a 100% cross-validation accuracy for demyelinating lesions and 99% cross-validation accuracy over the whole sample of lesions, glioblastomas, and astrocytomas (230). Similarly, analysis over multiple inputs has demonstrated superior accuracy over single-feature metrics in efforts to use patient demographics and existing magnetic resonance images to predict second attacks in clinically isolated syndrome (304). The addition of metabolite ratios among N-acetyl aspartate, choline, and creatine as measured by ^1^H-MRS slightly increased F1 score in linear discriminant analyses using age, disease duration, lesion load, and expanded disability status scale (EDSS) score to distinguish between clinically isolated syndrome and relapse-onset multiple sclerosis, between relapsing-remitting and primary progressive multiple sclerosis, and between relapsing-remitting and secondary progressive multiple sclerosis (305). With a continued explosion of free software libraries and packages enabling the straightforward implementation of a varied zoo of iterative classification and learning algorithms, like scikit-learn (306), PyTorch (307), TensorFlow (308), and others in Python and a number of packages in R (309), the influence of such approaches is likely to grow in coming years (310).

In the case of analysis pipelines that output intuitively interpretable models, like decision trees built on untransformed metabolite ratios, or classifiers that can be otherwise queried to determine the relative importance of particular features (311), some classification algorithms can not only offer practically useful diagnostic tools but also provide novel insights about the physiology of multiple sclerosis and reshape priorities regarding the metabolic targets of future investigations thereof. As with any analysis, however, the accuracy and generalizability of even the most sophisticated classifier depends on the precision and reproducibility, respectively, of its inputs. A systematically shorter *T*_2_ in white-matter metabolite resonances comprising the creatine signal of multiple sclerosis patients relative to control may, for example, enable a classifier to identify patients on the basis of higher apparent concentrations of creatine-referenced choline acquired from a long-*T*_*E*_ scan. Regardless of the potentially even perfect cross-validation accuracy achievable by this classifier, because its inputs are artefactually based on creatine *T*_2_, which disproportionately affects those metabolite signals referenced to creatine and acquired at long echo time, and not on molecular concentrations, which are theoretically invariant to such sequence parameters, such a classifier could not be confidently generalized until the source of the original between-group difference—creatine *T*_2_ and not choline concentration—were appreciated with precision and subsequent clinical applications limited accordingly.

Such an appreciation of the intricate web of factors upon which a metabolite concentration obtained via proton spectroscopy rests, however, depends on thorough understanding and careful control of many subtleties in ^1^H-MRS experiment design, acquisition and processing, and spectral quantitation, as well as their incidental or necessary alteration with multiple sclerosis disease state. In practice, this means offering and demanding not only commonly accepted but also evidence-based standards for spectroscopic data acquisition, analysis, and reporting (234). It also means evaluating new data with as much an eye to the potential methodological confounds of proton spectroscopy as to the novelty or biological plausibility of the putative findings at hand. With such an appreciation for the many methodological details overviewed in the present review, the march toward suitable diagnostic biomarkers for multiple sclerosis may be a slow one, perhaps occasionally punctuated by stepwise advances in the quality or throughput of ^1^H-MRS acquisitions or the information complexity enabled by techniques for extracting clinically useful features thereof. Greater characterization and understanding of each step taken, however, increases the capacity of the field to self-correct in a meaningful direction, ultimately maximizing the probability of safe and accurate multiple sclerosis diagnostic pipelines supported by information derived from ^1^H-MRS.

## Author Contributions

KS reviewed literature, tabulated data, performed statistical analysis, composed, edited, and approved manuscript. KL, DP, and CJ edited and approved manuscript.

### Conflict of Interest

The authors declare that the research was conducted in the absence of any commercial or financial relationships that could be construed as a potential conflict of interest.
